# Ultrasonic-Assisted Stewing Enhances Chicken Soup Flavor by Promoting Flavor Precursor Release and Key Aroma Compound Formation

**DOI:** 10.3390/foods15172978

**Published:** 2026-08-25

**Authors:** Dandan Zhang, Ziyan Yue, Ruiying Chen, Huanjuan Guo, Weikang Guo, Cuiping Feng, Yingchun Zhu

**Affiliations:** 1 Social Service Department of Shanxi Agricultural University, Taigu 030801, China; zhangdandan@sxau.edu.cn; 2 College of Food Science and Engineering, Shanxi Agricultural University, Taigu 030801, China; 15525794820@163.com (Z.Y.); 18734422667@163.com (R.C.); 13485436066@163.com (H.G.); 17835265718@163.com (W.G.); ndfcp@163.com (C.F.); 3 School of Food and Health, Beijing Technology and Business University, Beijing 100048, China

**Keywords:** chicken soup, ultrasonic-assisted stewing, volatile organic compounds, non-volatile compounds, flavor precursors

## Abstract

Chicken soup is widely appreciated for its nutritional value and flavor. This study investigated the effects of ultrasonic-assisted stewing (UAS) on flavor and non-volatile compounds (NVCs) using gas chromatography-mass spectrometry (GC-MS) and ultra-performance liquid chromatography-tandem mass spectrometry (UPLC-MS/MS) analysis. GC-MS identified 39 volatile organic compounds (VOCs). At 120 min of stewing, the total VOCs concentration in the UAS group reached 150.08 mg/kg, approximately 5.10-fold that of the control group (29.41 mg/kg). Multivariate analysis combined with odor activity values further screened 10 differential VOCs. Aroma recombination and omission tests confirmed that 7 key VOCs, including (E)-2-octenal and (E,E)-2,4-decadienal, contributed to the meaty and fatty sensory attributes. UPLC-MS/MS analysis detected 5032 NVCs across 20 categories, among which 322 key differential NVCs, including small peptides, fatty acids, and nucleotides, showed significant differences between the UAS and control groups. Overall, UAS enhanced the flavor complexity of chicken soup by facilitating the release of flavor precursors, including small peptides, nucleotides, and lipid-derived NVCs, and promoting the formation of key VOCs. This study provides theoretical and technical support for the application of UAS in soup processing.

## 1. Introduction

Chicken is a nutritious and affordable meat, rich in protein, fatty acids, vitamins B, and essential trace minerals [1]. Stewing is one of the most traditional and commonly used methods for chicken preparation. During stewing, chicken soup undergoes the release of volatile organic compounds (VOCs) and taste-active substances such as flavor peptides, nucleotides, and free amino acids, which lead to its enhanced palatability and support efficient digestion and absorption [2]. In traditional Chinese dietary culture, chicken soup is considered a nutritious tonic with potential therapeutic properties, commonly linked to the prevention of colds, the alleviation of inflammation, and the enhancement of immune function. It is particularly advised for children, seniors, and people with compromised health [3]. Chicken soup exhibits considerable potential for industrial applications due to its nutritional and flavor qualities. However, the traditional method of slow stewing, which is effective in extracting nutrients and flavor compounds, is limited by high time/energy demands and limited process controllability, hindering large-scale industrial production.

Ultrasound technology has attracted wide attention in the food industry owing to its eco-friendly, highly efficient, and energy-saving characteristics. It has been widely employed for the extraction of proteins, lipids, polysaccharides, and various bioactive compounds from food matrices [4]. Recently, ultrasonic technology has also been introduced into soup preparation, aiming to enhance product quality in terms of flavor, nutrition, and physical properties. Compared with conventional stewing methods, ultrasound-assisted stewing (UAS) significantly enhances stewing efficiency and achieves similar or superior results within a shorter time in terms of quality. This phenomenon is mainly attributed to the mechanical effects of ultrasound, including shear force, cavitation, and homogenization, which can effectively disrupt the microstructure of raw materials, release nutrients from within cells, and accelerate the migration and transformation of flavor precursor compounds [5]. For instance, Zhu et al. [6] employed thermal-ultrasound assisted processing to obtain Greenland halibut soup and found significant increases (*p* < 0.05) in nutritional components, notably the variety of fatty acids increased from 8 to 14 species, and their concentration rose to 14-fold higher compared with control group. Additionally, the average particle size of micro-nano particles (MNPs) in the soup decreased from 1582.28 nm to 605.92 nm, which enhanced the stability of the entire system. In chicken soup, Qi et al. [7] further demonstrated that UAS increased the lipid content from 0.3% to 1.2% and improved emulsion stability, with the total content of aroma-active compounds reaching 314.70 ng/mg after 1 h of thermo-ultrasound treatment. They suggested that the enhanced aroma intensity was associated with increased lipid release and improved emulsion stability, whereas prolonged ultrasound promoted lipid oxidation and reduced the retention of aroma-active compounds. Our previous research also showed that UAS markedly improved (*p* < 0.05) the nutritional quality of chicken soup [8]. UAS improved the rheological and emulsifying properties of chicken soup, resulting in enhanced stability and the formation of smaller and more uniform MNPs. Additionally, the concentration and variety of VOCs were elevated of chicken soup, further enhancing the flavor profiles [8]. In summary, ultrasound technology, particularly UAS, offers significant advantages in the preparation of soup products by promoting the migration of nutrients and enhancing flavor quality. However, the fundamental mechanisms of flavor formation in soup products, particularly the roles of key VOCs and precursor substances, remain insufficiently understood and therefore require further investigation.

Flavor is a critical determinant of chicken soup quality and plays a significant role in shaping consumer preferences. The flavor was mainly derived from VOCs, particularly aldehydes, alcohols, ketones, terpenes, and various heterocyclic compounds. The formation of these VOCs involves a complex interaction of lipid oxidation, Maillard reactions, thiamine breakdown, and thermal degradation. Moreover, secondary reactions might occur among the intermediates generated in these primary reactions, which further shape the flavor profile of chicken soup. Tian et al. [9] reported that the dominant aromatic qualities of fish soup were meaty and greasy, which were strongly associated with lipid oxidation, especially the oxidation of unsaturated triglycerides. Although conventional analytical techniques have identified various flavor-related compounds, they often provide a fragmented view and fail to capture the dynamic interactions within metabolic networks. Recently, ultra-performance liquid chromatography-tandem mass spectrometry (UPLC-MS/MS) has become a valuable method for the thorough analysis of small-molecule non-volatile compounds (NVCs) [10]. UPLC-MS/MS provides a broad analytical strategy by simultaneously profiling diverse metabolite signatures and expanding the coverage of detectable compounds [11]. Consequently, UPLC-MS/MS has been increasingly applied to investigate flavor formation, offering new opportunities to identify key precursor molecules associated with characteristic flavor attributes.

Despite these advances, the relationship between non-volatile flavor precursors and key aroma-active compounds during UAS remains unclear. We hypothesized that UAS promotes the release and transformation of flavor precursors through cavitation and mechanical effects, thereby enhancing the formation of non-volatile flavor compounds and key aroma-active compounds. To test this hypothesis, the aroma profile of UAS chicken soup was characterized using gas chromatography-mass spectrometry (GC-MS), which also facilitated the identification of key VOCs. The impact of these VOCs on the overall aroma profile was assessed by calculating their odor activity values (OAVs). To confirm the contribution of key aroma-active compounds, aroma recombination and omission tests were further conducted. Furthermore, ultra-performance liquid chromatography-tandem mass spectrometry (UPLC-MS/MS) was employed to identify NVCs in chicken soup and to analyze the dynamic variations of small-molecule NVCs throughout the stewing process. Potential precursor compounds contributing to the formation of aroma-active volatile compounds were also identified. This research aims to enhance understanding of the flavor characteristics of chicken soup after UAS. It also offers a theoretical foundation for managing flavor through nutrition and for the development of high-quality soup products.

## 2. Materials and Methods

### 2.1. Experiment Materials

The three-yellow chickens of the same breed and batch, aged 300 days, were procured from Donghai Market (Jinzhong, China) to ensure sample consistency. Additionally, the chickens used in the experiment were comparable in shape, size, and weight. The chicken carcasses were immediately preserved at −2 °C until subsequent processing. Seasoning materials, including salt, ginger, and scallion, were procured from Jiajiali Supermarket (Jinzhong, China) and confirmed to comply with food-grade quality requirements. Authentic aroma standards, including (E)-2-hexenal (purity 98.0%), (E,E)-2,4-hexadienal (purity 80.0%), and (E,E)-2,4-heptadienal (purity 90.0%), etc., were purchased from Macklin (Shanghai, China). 1,2-Dichlorobenzene (purity 98.0%; Macklin, Shanghai, China) was used as the internal standard. A mixture of n-alkanes (C8–C30) was purchased from Sigma-Aldrich Co. (St. Louis, MO, USA). HPLC-grade methanol and acetonitrile were obtained from CNW Technologies GmbH (Bielefeld, Germany), and LC-MS-grade acetic acid was purchased from Thermo Fisher Scientific (Shanghai, China). All other analytical-grade reagents were obtained from Sinopharm Chemical Reagent Co., Ltd. (Shanghai, China).

### 2.2. Preparation of Chicken Soup

After thorough washing, the chicken carcasses were cut into uniform cubes (3.0 ± 2 cm^3^) and thoroughly mixed. The precooked chicken nuggets and purified water (200 g:600 g) were sealed in heat-resistant plastic bags designed for high-temperature cooking. In the control group, the sample bags were placed in a stewing pot (THC-1000SF, Tianhua Ultrasonic Electronic Instrument, Jining, China), heated to 95 °C, and maintained at this temperature for 2 h without ultrasonic assistance. In the UAS group, ultrasonic treatment (frequency: 24.5 kHz, power: 800 W) and stewing were simultaneously initiated at 95 °C and continued for 2 h. The stewing pot was equipped with an automatic thermostatic temperature-control system, which maintained the preset temperature throughout the stewing process. The temperature was automatically regulated and maintained at 95 °C throughout the treatment. The ultrasound was applied in cycles of 30 min, consisting of 20 min of operation followed by 10 min of pause, according to the equipment instructions. Samples from both the control and UAS groups were collected at 0, 30, 60, 90, and 120 min. The samples were labeled as C0–C120 for the control group and U0–U120 for the UAS group, respectively. After cooling to room temperature in an ice-water bath, the samples were filtered through gauze to remove chicken nuggets and other impurities, yielding the final chicken soup samples.

### 2.3. Sensory Evaluation

Sensory evaluation was performed according to the procedure described by Wei et al. [12], with slight modifications. In brief, a panel of 10 individuals (four males and six females) aged 20–50 years, all with substantial experience in quantitative descriptive analysis, participated in the evaluation. The panelists were familiarized with the definitions of the five target aroma attributes, their corresponding reference solutions, and the 10-point intensity scale before formal evaluation. The odor attributes, including chickeny, meaty, fatty, mushroom, and sweet notes, together with their perceived intensities, were assessed by comparison with standard solutions of ethanol-diluted (E,E)-2,4-decadienal, (E,Z)-2,4-decadienal, (E,E)-2,4-heptadienal, 1-octen-3-ol, and (E)-2-heptenal prepared at different concentrations. All samples were presented in 50 mL vials, each labeled with a randomly generated three-digit code to ensure blinding. The panelists evaluated both odor quality and intensity using a 10-point scale, where a score of 1 represented the absence of the attribute and 10 denoted an extremely strong perception. To prevent olfactory fatigue, panelists were required to inhale fresh air for 20 s after each smelling test. The sensory evaluation was conducted in the laboratory of the College of Food Science and Engineering at Shanxi Agricultural University (Jinzhong, China). All participants signed an informed consent before the sensory evaluation. The rights and privacy of all participants were protected during the execution of the study.

### 2.4. Volatile Organic Compounds Analysis

The VOCs in chicken soup were analyzed using a Hexin 1000 GC-MS instrument (Hexin Instrument Co., Ltd., Guangzhou, China) equipped with a 30 m × 0.25 mm × 0.25 µm MEGA-5MS capillary column (Restek Corporation, Bellefonte, PA, USA). SPME-GC-MS analysis was conducted according to the method described by Bai et al. [13], with slight modifications. 7 g of chicken soup were mixed with 1 g of sodium chloride and sealed in a 20 mL headspace vial equipped with a polytetrafluoroethylene (PTFE) septum. A 1,2-dichlorobenzene solution (3.26 mg/mL, prepared in ethanol) was added and agitated in each vial. Next, the samples were equilibrated in a water bath at 80 °C for 30 min, and the headspace volatiles were extracted using a 50 μm polydimethylsiloxane/divinylbenzene (PDMS/DVB) fiber (Supelco, Inc., Bellefonte, PA, USA). The extracted volatiles were first desorbed at 250 °C for 5 min in splitless mode at the GC injection port. Helium was used as the carrier gas at a constant flow rate of 0.8 mL/min. The GC oven temperature program started at 40 °C (held for 4 min), increased to 230 °C at a rate of 5 °C/min, and was held at the final temperature for 4 min. The MS was operated in electron impact (EI) ionization mode at 70 eV, with a scan range of m/z 30–350 and the ion source temperature set to 250 °C. VOCs were identified by comparing their mass spectra with those in the NIST 2.0 library. Only compounds with a NIST 2.0 library (National Institute of Standards and Technology, Gaithersburg, MD, USA) matching score greater than 70% were retained for subsequent analysis. Retention indices (RIs) were calculated using n-alkane standards (C8–C30) under identical GC conditions to further support compound identification. The identities of selected compounds were further confirmed by comparing their retention times and fragmentation patterns with those of authentic standards under identical GC-MS conditions. The relative concentration of each VOC was calculated by comparing the peak area of the compound to that of the internal standard, using the following formula:

$$C=\frac{A_{x}\times C_{0}\times V\times 1000}{A_{0}\times m}\;(mg/kg)$$

where *C* and *C*_0_ represented the concentration of volatile compounds (mg/kg) and internal standard (mg/mL), respectively; *Ax* and *A*_0_ indicated the peak area of volatile compounds and internal standard, respectively; *V* represented the volume of internal standard added to the sample (mL); and *m* represented the mass of sample (g).

The odor activity value (*OAV*) reflects the contribution of different VOCs to the overall aroma of a sample. It is defined as the ratio of the concentration of a VOC to its odor threshold in water, and is calculated as follows:

$$OAV=\frac{C}{T}$$

where *OAV* is the odor activity value of the VOCs; *C* is the concentration of the VOCs (mg/kg); *T* is the odor threshold of the VOCs in water (mg/kg).

### 2.5. Aroma Recombination and Omission Experiments

The preparation of the odorless matrix was based on the method described by Wang et al. [14]. Odor-active compounds were removed by repeated extraction of the chicken soup using trichloroacetic acid. To construct the aroma recombination model, 5 g of deodorized chicken soup was transferred into a 15 mL headspace vial, and 10 VOCs that were identified that significantly influencing the overall aroma profile were added to the sample at concentrations determined by GC-MS. After thorough mixing and equilibration for 10 min, sensory evaluation was performed to compare the aroma characteristics of the recombined sample with those of the original chicken soup. An aroma omission test was conducted to evaluate the actual contribution of each VOC to the overall flavor profile. Each of the 10 VOCs in the recombination model was individually omitted, then assigned a random number prior to sensory evaluation. Omission tests were conducted by comparing each omission model with the complete recombination model using a triangle test. During evaluation, panelists were instructed to identify the sample that differed from the other two [15].

### 2.6. UPLC-MS/MS Analysis

UPLC-MS/MS analysis was performed as previously described Liu et al. [16] with minor modifications. Samples stored at −80 °C were thawed on ice and vortexed for 10 s. An extraction solvent (acetonitrile:methanol = 1:4, *v*/*v*) containing the internal standard (200 μL) was then added to 200 μL of the sample. After vortexing for 3 min, the mixture was centrifuged at 15,616× *g* for 10 min at 4 °C. A 350 μL aliquot of the supernatant was collected and fully concentrated. The residue was reconstituted in 100 μL of methanol:water (7:3, *v*/*v*), vortexed for 3 min, and subsequently sonicated for 10 min in an ice-water bath. The sample was centrifuged at 15,616× *g* for 3 min at 4 °C. An 80 μL aliquot of the supernatant was then collected for LC-MS analysis. LC-MS/MS analysis was conducted using an ultra-performance liquid chromatography (UPLC) system (SCIEX, Warrington, UK) connected to a TripleTOF 5600 Plus high-resolution tandem mass spectrometer (SCIEX, Warrington, UK). The separation was performed on an ACQUITY UPLC T3 column (100 mm × 2.1 mm, 1.8 μm; Waters Corporation, Milford, MA, USA). Quality-control (QC) samples were included in the analytical sequence to monitor the stability and reproducibility of the LC-MS/MS system. Analytical stability was assessed from the consistency of total ion chromatograms (TICs), peak responses, and retention times across QC samples.

Raw mass-spectrometric data were converted to mzXML format using ProteoWizard (version 3.0). Peak detection, peak alignment, and retention-time correction were performed using XCMS (version 3.18.0). Peak areas were corrected using a support vector regression (SVR)-based procedure, and features with missing values in more than 50% of the samples were excluded from subsequent analysis. NVC annotation was performed using an in-house database, integrated public databases, predictive databases, and the metDNA platform. HMDB 5.0 and KEGG databases were additionally used to verify the annotated compounds. The processed data matrix was then used for multivariate statistical analysis.

### 2.7. Statistical Analysis

Three independently prepared chicken soup samples were used as biological replicates for each treatment (*n* = 3), and the results are expressed as the mean ± standard deviation (SD). Statistical analyses were performed using the Statistica software package (version 8.1, StatSoft Inc., Tulsa, OK, USA). Differences among multiple groups were evaluated by analysis of variance (ANOVA), followed by the least significant difference (LSD) post hoc multiple-comparison test when significant differences were detected. For pairwise comparisons between the UAS and control groups, Student’s *t*-test was used. Differences were considered statistically significant at *p* < 0.05. Multivariate analyses, including principal component analysis (PCA), orthogonal partial least squares discriminant analysis (OPLS-DA), and volcano plot analysis, were conducted using MetaboAnalyst 6.0 and Metware Cloud platform (https://cloud.metware.cn). The robustness of the OPLS-DA model was evaluated using 1000 permutation tests. Differential NVCs were screened using the combined criteria of VIP > 1, FC ≥ 2 or ≤0.5, and *p* < 0.05. Data processing and visualization were carried out using Origin 2024 and TBtools (version 2.096).

## 3. Results and Discussion

### 3.1. Analysis of Sensory Evaluation of Chicken Soup Under Different Stewing Methods

**Figure 1:**
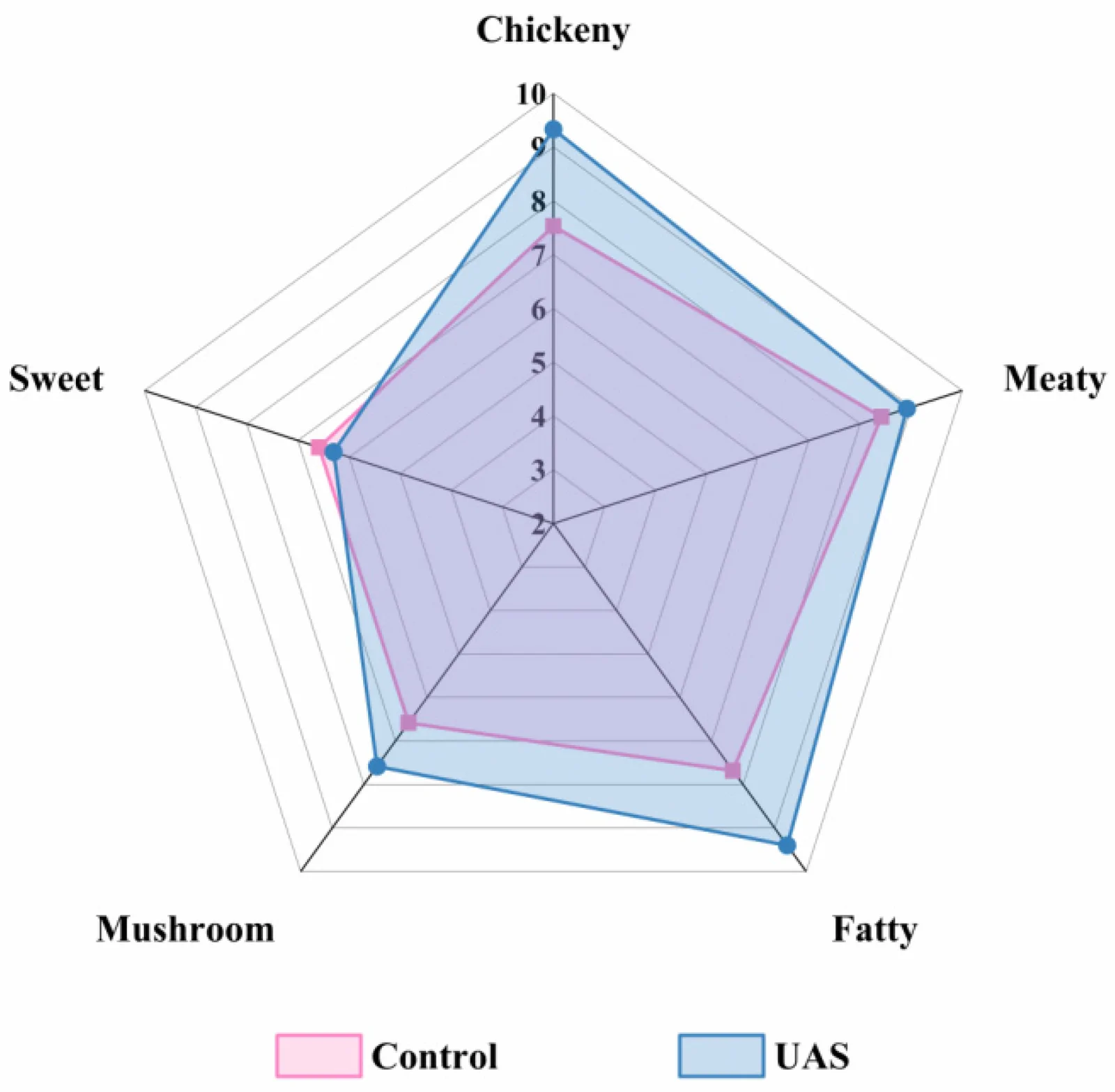
Analysis of sensory evaluation of chicken soup of different stewing method.

Sensory evaluation of aroma was performed for the UAS and control chicken soup samples using five sensory descriptors: chickeny aroma, meaty aroma, fatty aroma, mushroom aroma, and sweet aroma. Figure 1 shows the sensory evaluation of chicken soup prepared using different stewing methods. As shown in Figure 1, although there was no significant difference in the overall aroma intensity between the UAS group and the control group (*p* > 0.05), the UAS group exhibited higher intensities of chickeny, fatty, mushroom, and meaty aromas, while both groups showed comparable levels of sweet aroma. Overall, the sensory evaluation of the UAS group was superior to that of the control group. The flavor of chicken soup results from the interplay between taste and aroma, which originate from a complex mixture of non-volatile and volatile compounds. Under heating conditions, chicken underwent a series of chemical reactions in a short period of time, including lipid hydrolysis, lipid oxidation, and Maillard reactions, which subsequently led to the formation of a large number of VOCs [17]. Ultrasound treatment promoted faster hydrolysis of lipids into free fatty acids through cavitation effects and mechanical vibrations, thereby accelerating oxidation reactions and Maillard reactions. This process facilitated the generation of VOCs, such as aldehydes, ketones, pyrazines, and furans, which contributed to fatty, meaty, and soup-like aromas [18]. Meanwhile, ultrasound enhanced mass and heat transfer efficiency, leading to a more uniform distribution of flavor compounds throughout the soup and thereby improving the overall flavor profiles. The aroma attributes of VOCs in chicken soups prepared using the two different stewing methods varied, which may be attributed to variations in the composition and concentrations of the compounds. Therefore, further analysis and validation using instrumental techniques combined with multivariate statistical analysis were required.

### 3.2. Analysis of the VOCs of Chicken Soup Under Different Treatment Methods

#### 3.2.1. Analysis of the Dynamic Variation of VOCs of Chicken Soup Under Different Treatment Methods

**Figure 2:**
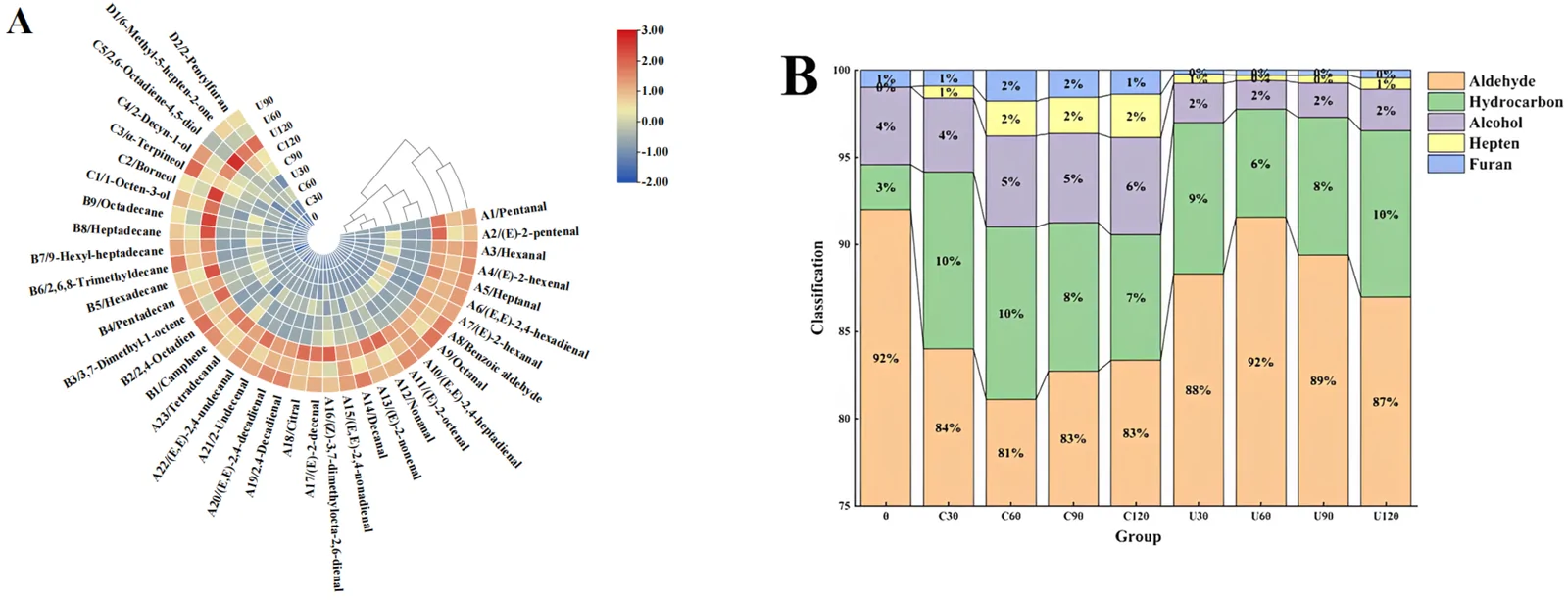
Analysis of the volatile organic compounds of chicken soup of different treatment method: (**A**): Heat map obtained from cluster analysis, (**B**): Percentage of VOCs of different categories plot. Notes: (**A**): Different colors represent different concentrations. (green = low, yellow = moderate, red = high).

The concentrations and compositions of VOCs in chicken soup were influenced by multiple factors, including the raw material composition, stewing time, stewing temperature, and stewing method [19]. As shown in Table S1 and Figure 2A, SPME-GC-MS analysis identified 39 VOCs in the chicken soup samples, comprising 23 aldehydes, 9 hydrocarbons, 5 alcohols, and 2 compounds classified as others. As the stewing time was extended, the VOCs concentration in chicken soups prepared by both stewing methods increased significantly (*p* < 0.05). The VOCs concentrations in the chicken soup from the UAS group and the control group at 120 min were 150.08 mg/kg and 29.41 mg/kg, respectively, and both values were significantly higher than those at 0 min (11.24 mg/kg, *p* < 0.05). Moreover, the VOC compositions in the chicken soups from both the UAS and control groups were generally similar, whereas their concentrations differed significantly. The concentrations of VOCs in the chicken soup of the UAS group at 30 min was 56.19 mg/kg, which was significantly higher than that in the control group at 120 min (29.41 mg/kg, *p* < 0.05). This difference was primarily attributed to the cavitation and thermal effects of ultrasound, which enhanced Maillard reactions, amino acid degradation, and lipid oxidation, thereby resulting in a markedly higher concentration of VOCs in the UAS group compared with the control group [20].

Mechanistically, acoustic cavitation can generate intense shear, microstreaming, and transient high-temperature/high-pressure microenvironments, which disrupt muscle and adipose structures and increase the release and dispersion of lipid- and protein-derived flavor precursors [7,21]. The greater exposure and dispersion of unsaturated lipids can favor hydroperoxide formation and cleavage, while enhanced mixing increases contact between amino compounds and reducing sugars [22,23]. Together, these effects promote lipid oxidation, Strecker degradation, and Maillard reactions, thereby facilitating the formation of aroma-active aldehydes, alcohols, ketones, and furans [18,24].

Aldehydes were the primary contributors to the flavor of chicken soup, and they were mainly formed through the oxidative degradation of lipids. Aldehydes comprised a substantial fraction of the VOCs in both the UAS and control groups, accounting for over 80% of the total VOC concentration (Figure 2B). The concentration of aldehyde compounds gradually increased with prolonged stewing time. Prolonged heating enhanced lipid oxidative degradation and promoted Strecker degradation reactions, resulting in an elevated concentration of aldehydes [25]. The aldehydes identified in chicken soup mainly included hexanal, heptanal, (E)-2-heptenal, octanal, (E)-2-octenal, nonanal, (E)-2-nonenal, decanal, (E,E)-2,4-nonadienal, (E)-2-decenal, (E,Z)-2,4-decadienal, (E,E)-2,4-decadienal, and (E,E)-2,4-undecenal. Among them, hexanal was an oxidation product of linoleic acid and arachidonic acid, while heptanal was derived from the degradation of linoleic acid and oleic acid [26]. (E,E)-2,4-Decadienal was a major secondary oxidation product of linoleic acid and was considered a key characteristic component contributing to the flavor of chicken. Huang et al. [27] found that the majority of straight-chain aldehydes were derived from the oxidation of unsaturated fatty acids, whereas branched-chain aldehydes were generated via the thermal degradation of amino acids such as valine, leucine, and isoleucine during the stewing process. After ultrasonic treatment, both the diversity and concentration of aldehydes in chicken soup were elevated. The concentration of aldehydes in the UAS 90 min group reached a maximum of 131.80 mg/kg, indicating that ultrasonic treatment could promote lipid oxidation, thereby promoting aldehyde formation [7]. The decrease in aldehyde concentration observed in the UAS group at 120 min could be attributed to the prolonged ultrasonic treatment, which could have led to the further degradation of aldehydes.

Hydrocarbons, mainly alkanes and alkenes, were important contributors to the VOCs in chicken soup. The concentration of hydrocarbons showed an increasing trend during the stewing process (Table S1 and Figure 2A). Hydrocarbons were the second most abundant compounds in chicken soup after aldehydes, mainly originating from fatty acid alkoxy radical decomposition and the addition of spices. Long-chain alkanes were mainly derived from the breakdown of fatty acid alkoxy radicals, while alkenes originated from the spices added during the stewing process [28]. Ultrasonic treatment significantly increased the concentration of hydrocarbons, reaching a maximum of 14.34 mg/kg in the UAS group at 120 min. This increase could be attributed to the promotion of decarboxylation and cleavage reactions of long-chain fatty acids by ultrasound. Camphene had a camphor aroma and was mainly derived from spices such as ginger [28]. Due to their relatively high odor thresholds, alkanes play a minor role in determining the overall flavor of chicken soup [29]. However, research has shown that the combined effect of various alkanes and alkenes can enhance the overall flavor of meat products [30].

Alcohols in chicken soup primarily arose from chemical degradation and the oxidation of fatty acids. Owing to their relatively high flavor thresholds, these alcohols contributed less significantly to the overall flavor profile than other VOCs, including aldehydes. As stewing time increased, prolonged heating promoted alcohol formation through aldehyde oxidation and triglyceride breakdown by facilitating chemical degradation and fatty acid oxidation, thereby leading to a gradual rise in alcohol concentration [31]. Among them, saturated alcohols contributed less to the flavor profiles, while unsaturated alcohols contributed more significantly due to their lower odor thresholds and more distinctive aroma characteristics [32]. At 120 min, the concentration of alcohol compounds in the UAS group (14.34 mg/kg) was significantly higher (*p* < 0.05) than that in the control group (1.64 mg/kg), indicating that ultrasonic treatment further promoted the generation of alcoholic compounds. 1-Octen-3-ol, derived from the oxidation of arachidonic, linoleic, and linolenic acids, is a major alcohol responsible for the characteristic mushroom-like aroma in chicken [29]. At 120 min, the concentration of 1-octen-3-ol in the UAS group (0.63 mg/kg) was significantly higher (*p* < 0.05) than that in the control group (0.15 mg/kg), suggesting that ultrasonic treatment facilitated the oxidation of fatty acids, particularly arachidonic and linolenic acids.

In addition to the above-mentioned aldehydes, hydrocarbons, and alcohols, small quantities of furans and ketones were also detected. Furan was the most important heterocyclic compound in chicken soup. 2-Pentylfuran, generated via the oxidation of linoleic acid and other n-6 polyunsaturated fatty acids, was an important contributor to the overall flavor profile of chicken soup [20]. The concentration of 2-pentylfuran progressively increased with extended stewing time, rising from 0.11 mg/kg to 0.41 mg/kg (control group, 120 min) and 0.67 mg/kg (UAS group, 120 min). This demonstrated that linoleic acid and other fatty acids in chicken underwent oxidative degradation during prolonged heating, thereby promoting 2-pentylfuran formation. Ketones were important precursors for the formation of fatty flavors and were typically generated through β-oxidation of fatty acids [33]. 6-Methyl-5-hepten-2-one, characterized by animal fat-like aroma notes, was mainly generated via the decarboxylation of β-keto acids formed during saturated fatty acid β-oxidation [34]. The concentration of 6-methyl-5-hepten-2-one increased progressively during thermal processing, reaching 0.73 mg/kg in the control group and 0.99 mg/kg in the UAS group at 120 min, indicating that ultrasonic treatment facilitated the oxidative degradation of polyunsaturated fatty acids, thereby promoting the formation of ketone compounds.

#### 3.2.2. Analysis of Differences in VOCs of Chicken Soup Under Different Treatment Methods

PCA and OPLS-DA were used to evaluate differences in VOC profiles between the UAS and control groups. PCA showed clear separation between the two groups, with PC1 and PC2 explaining 77.7% and 7.4% of the total variance, respectively (Figure S1A). OPLS-DA further confirmed this separation, with R^2^X = 0.622, R^2^Y = 0.801, and Q^2^ = 0.796 (Figure S1B). The permutation test further supported the robustness of the model (Figure S1C). These results indicate distinct differences in VOC composition between the two stewing methods.

Variable importance in projection (VIP) scores in the OPLS-DA model were computed to assess the contribution of individual VOCs to sample discrimination. Compounds with VIP values greater than 1 are considered important for group discrimination, with higher VIP values indicating greater differences among samples [35]. Figure S1D presented the VIP score analysis of the UAS group and control group, 23 VOCs with VIP values > 1 were identified. These VOCs included: (E,E)-2,4-hexadienal, 2,4-Octadien, (E)-2-heptenal, (E)-2-pentenal, 2,6,8-Trimethyldecane, (E,E)-2,4-decadienal, (E,E)-2,4-heptadienal, citral, (E)-2-nonenal, (E)-2-octenal, hexanal and 1-octen-3-ol and so on. These VOCs served as discriminant markers for distinguishing chicken soups prepared by UAS stewing or usual stewing.

#### 3.2.3. Analysis of Key Differential VOCs of Chicken Soup Under Different Treatment Methods by OAV

Aroma profiles were evaluated based on both the concentrations of VOCs and their respective odor thresholds. Compounds with OAVs ≥ 1 were defined as aroma-active, as they significantly contributed to the overall flavor profile, and higher OAVs corresponded to greater contributions to aroma [36]. The varieties and concentrations of VOCs in chicken soup exhibited significant variations depending on different stewing methods and times (Table 1). In the chicken soup, 26 VOCs with OAVs ≥ 1 were detected, comprising 21 aldehydes, 3 alcohols, 1 furan, and 1 ketone. The VOCs contributed substantially to the overall aroma profile, with compounds exhibiting OAVs ≥ 100 defined as key aroma compounds that determined the primary odor characteristics. The control group (120 min) contained 12 key VOCs (OAV ≥ 100): hexanal, heptanal, octanal, (E)-2-octenal, nonanal, (E)-2-nonenal, decanal, (E)-2-decenal, (E,Z)-2,4-decadienal, (E,E)-2,4-undecanal, 1-octen-3-ol, and 2-pentylfuran. The UAS group (120 min) contained 15 key aroma compounds, including hexanal, heptanal, (E)-2-heptenal, octanal, (E)-2-octenal, nonanal, (E)-2-nonenal, decanal, (E,E)-2,4-nonadienal, (E)-2-decenal, (E,Z)-2,4-decadienal, (E,E)-2,4-decadienal, and (E,E)-2,4-undecadienal, 1-octen-3-ol, and 2-pentylfuran. The key aroma compounds were identical among U60, U90, and U120 samples, while their OAV values progressively increased with extended ultrasonic time, indicating similar aroma characteristics during the stewing process.

The identified key aroma compounds were mainly composed of aldehydes, alcohols, and furans. Of these compounds, aldehydes were regarded as the predominant VOCs in the meat products, most of which exhibited low odor thresholds and significant contribution to flavor profiles. In chicken soup, short-chain aliphatic aldehydes, such as hexanal, heptanal, octanal, and nonanal, typically imparted fruity, green, or grassy notes [32]. Compounds classified as trans-alkenals, such as (E)-2-nonenal, (E)-2-decenal, and (E,E)-2,4-decadienal, generally contribute fatty and meaty odor notes [37]. Aromatic aldehydes typically imparted almond or caramel aromas, such as benzoic aldehyde. Therefore, aldehydes, as the predominant VOCs formed during the stewing process of chicken soup, significantly contribute to the characteristic fatty, meaty, and fruity aromas. Alcohols, characterized by their low odor thresholds, also contributed significantly to the characteristic flavor profiles in chicken soup. 1-Octen-3-ol contributed a mushroom aroma, exhibited concentrations that were significantly influenced by the stewing methods employed. At 120 min, the OAV of 1-octen-3-ol in the UAS group (15,057.51) was roughly five-fold higher than that in the control group (3075.46), contributing a characteristic aroma to the chicken soup. The concentration of 2-pentylfuran progressively increased during stewing, playing a significant role in imparting nutty and fruity notes to the chicken soup.

#### 3.2.4. Screening and Analysis of Key Differential VOCs of Chicken Soup Under Different Stewing Methods

Based on multidimensional screening criteria, this study defined key differential VOCs as those meeting all the following conditions: VIP > 1, absolute fold change (FC) > 5, *p* < 0.05, and OAV ≥ 1 [38]. As shown in Figure S2, ten key differential VOCs were identified, including (E)-2-hexenal, (E,E)-2,4-hexadienal, (E)-2-heptenal, benzoic aldehyde, 1-octen-3-ol, (E,E)-2,4-heptadienal, (E)-2-octenal, (E,Z)-2,4-decadienal, (E,E)-2,4-decadienal, (E,E)-2,4-undecanal. These compounds are key contributors to the overall aroma profile of chicken soup.

**Figure 3:**
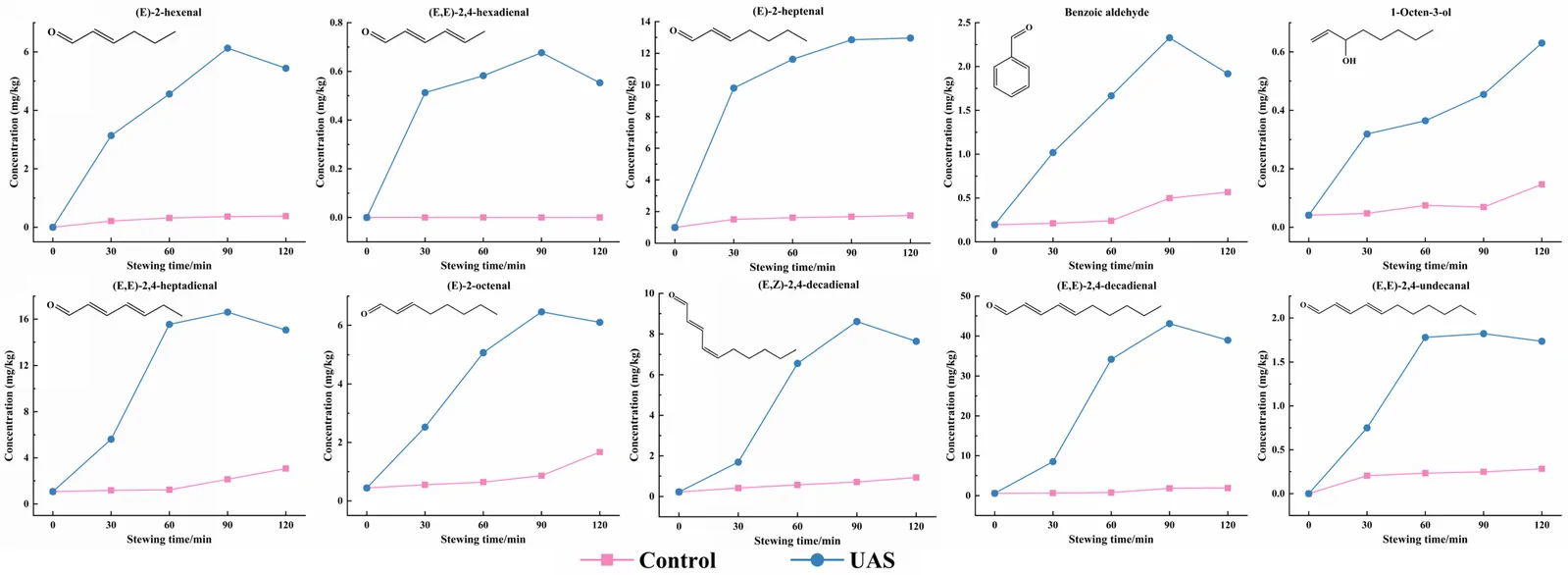
Concentration profiles of key differential volatile organic compounds in chicken soup under different stewing methods.

Figure 3 illustrated the variation trends of the key differential VOCs in UAS and control chicken soup. As shown in Figure 3, the UAS chicken soup contained 10 key differential VOCs, whereas the control group chicken soup contained 9 key differential VOCs, with (E,E)-2,4-hexadienal being absent. The concentrations of key differential VOCs in the UAS group chicken soup were significantly higher (*p* < 0.05) than those in the control group. After 90 min of ultrasonic treatment, most aldehydes exhibited higher concentrations in the UAS chicken soup than in the control group, suggesting that ultrasonic processing promoted lipid oxidation, which was the primary synthetic pathway for straight-chain aldehydes [39]. Among these compounds, (E,E)-2,4-decadienal was the richest key differential VOC. Derived from the secondary oxidation of linoleic acid, it was the principal contributor to the fatty aroma of the chicken soup [40]. Other aldehydes exhibited aromas such as fatty, chicken, and meaty notes. However, excessive ultrasonic treatment (120 min) might lead to further oxidative degradation of some aldehydes, resulting in a decrease in their concentrations. In addition, (E,E)-2,4-hexadienal was a key differential VOC, it was only detected in the UAS group, indicating that ultrasonic treatment not only significantly increased the concentration of VOCs in chicken soup (*p* < 0.05), but also enhanced the diversity of VOCs. 1-Octen-3-ol was derived from the oxidation of linoleic acid and arachidonic acid, possesses characteristic mushroom-like and meaty aromas. With prolonged ultrasound time, the concentration of 1-octen-3-ol exhibited a continuous and significant increase (*p* < 0.05), attributable to enhanced oxidation of lipids and unsaturated fatty acids driven by ultrasonic cavitation effects, which consequently promoted the formation of this aroma compound [21].

#### 3.2.5. Identification of Key Characteristic VOCs in Chicken Soup Using Recombination-Omission Tests

**Figure 4:**
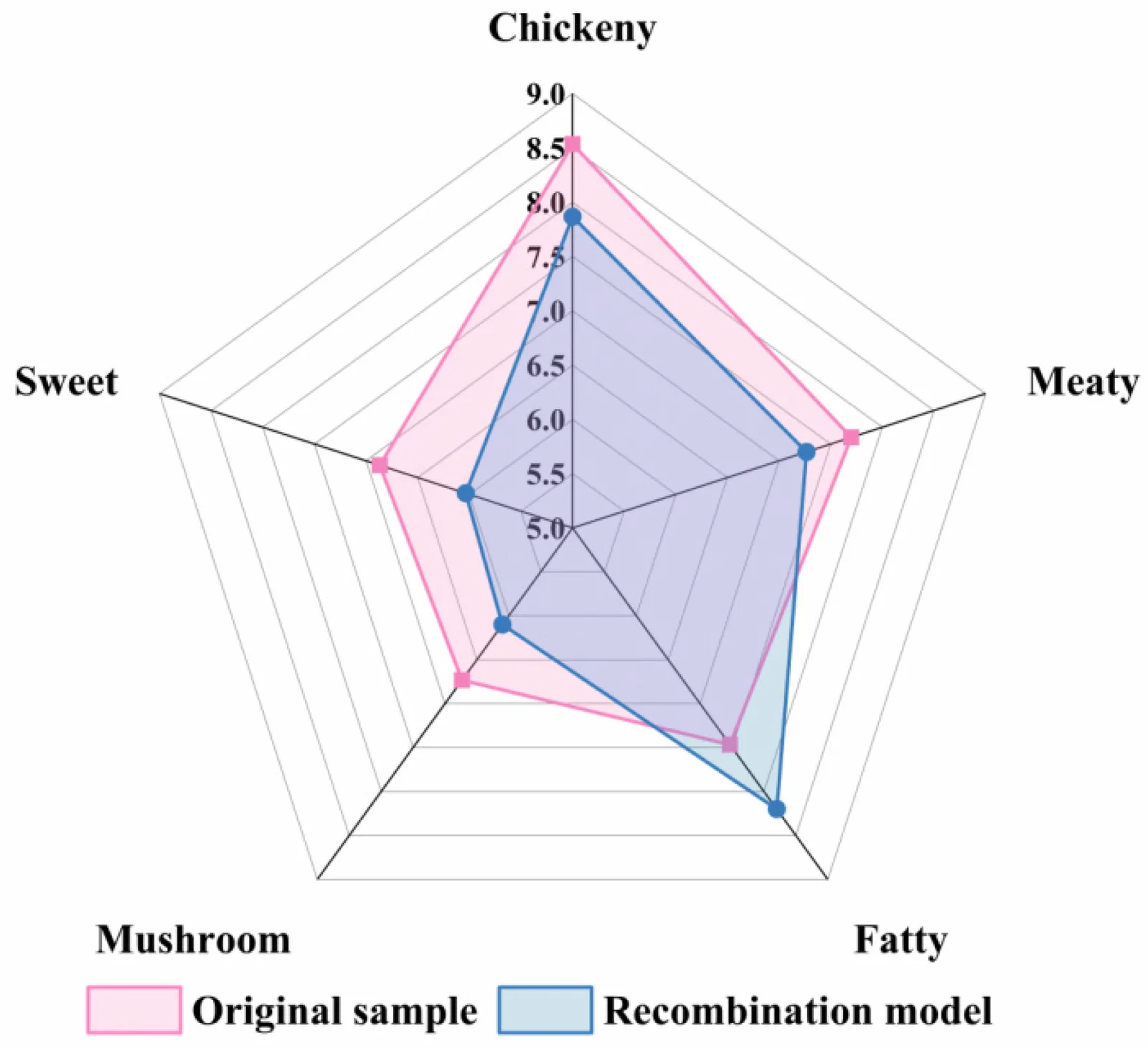
Analysis of aroma profiles in the original sample and recombinant model.

Triangle tests and sensory evaluations were conducted to assess the contributions of 10 key differential VOCs to the overall aroma of chicken soup by comparing the recombination model with the original samples. As shown in Figure 4, the aroma profiles of the recombination model exhibited high similarity to that of the original chicken soup. The recombination model exhibited slightly lower chickeny, meaty, mushroom, and sweet aroma intensities compared to the original samples;but these differences were not statistically significant (*p* > 0.05). However, due to the influence of aldehydes with fatty aromas such as (E)-2-octenal, (E,Z)-2,4-decadienal, and (E,E)-2,4-decadienal, the fatty aroma in the recombination model was slightly higher than that of the original samples. This difference could be attributed to the impact of the standard reagents on the fatty aroma of the recombined sample. The differences between the recombination model and the original samples could be attributed to the incomplete identification of compounds with low odor activity values, matrix effects, or the omission of compounds that played auxiliary roles in the overall aroma during the recombination process [15].

Aroma omission tests were conducted to evaluate the roles of individual compounds in shaping the overall aroma profile of chicken soup. In this test, certain individual or group of aroma compounds were removed from the recombination model to generate various omission models. These omission tests were then evaluated against the complete recombination model using a triangle test to identify perceptible differences [41]. The experimental design and results of the omission models were presented in Table 2. Among the 10 omission tests, 7 omission models showed significant differences compared to the recombination model (*p* < 0.05). Among them, 4 omission models exhibited the highest significant differences compared to the recombination model (*p* < 0.01), and 3 omission models showed the higher significant differences (*p* < 0.001). In summary, 7 VOCs in the recombination model were identified as critical contributors to maintaining its overall aroma profiles. The absence of these compounds induced marked alterations, resulting in statistically significant differences from the complete recombination model in sensory evaluations. The omission models lacking (E,E)-2,4-hexadienal, (E)-2-octenal, or (E,E)-2,4-decadienal showed extremely significant differences compared to the recombination model (*p* < 0.001). Most sensory panelists were able to clearly identify the aroma differences caused by these omissions. These differences were mainly reflected in the loss of chickeny, fatty, and sweet aroma characteristics in the omission models. Additionally, the omission models lacking the above three compounds, as well as the omission model missing (E,E)-2,4-heptadienal which contributed fatty and chickeny aromas showed highly significant differences compared to the recombination model (*p* < 0.01). The omission models lacking (E)-2-hexenal, (E)-2-heptenal, and (E,Z)-2,4-decadienal exhibited significant alterations in spicy and fruity aroma, demonstrating statistically significant differences (*p* < 0.05) from the complete recombination model. These results suggest that the majority of aldehydes contribute substantially to the overall aroma profile of chicken soup. Furthermore, the omission models lacking benzoic aldehyde, 1-octen-3-ol, or (E,E)-2,4-undecadienal showed no significant differences (*p* > 0.05) from the complete recombination model. This could be due to their relatively low concentrations in chicken soup resulting in insignificant aroma impact. Additionally, differences between the odorless matrix and the original sample might have influenced the volatility and perception of these aroma compounds [42]. Therefore, this experiment confirmed that (E,E)-2,4-hexadienal, (E)-2-octenal, (E,E)-2,4-decadienal, (E,E)-2,4-heptadienal, (E)-2-hexenal, (E)-2-heptenal, and (E,Z)-2,4-decadienal were key characteristic VOCs in chicken soup.

Notably, although 1-octen-3-ol showed a high OAV, its omission did not produce a significant sensory difference (*p* > 0.05), indicating that a high OAV does not necessarily correspond to an indispensable sensory role in a complex aroma mixture. Its contribution may have been partially compensated by other odorants or modulated by matrix effects, as perceptual interactions among odorants are concentration- and matrix-dependent [15]. Conversely, the significant omission effects observed for several aldehydes demonstrate that their sensory importance was confirmed in the mixture context rather than inferred solely from their individual OAVs [41,42].

Moreover, sensory evaluation further confirmed that the UAS group exhibited higher intensities of chickeny, meaty, and fatty aromas, which were consistent with the increased contributions of key aldehydes, particularly (E)-2-octenal, (E,Z)-2,4-decadienal, (E,E)-2,4-decadienal, and (E,E)-2,4-heptadienal. The enhanced mushroom aroma was also consistent with the increased level of 1-octen-3-ol. These sensory–chemical associations were further confirmed by aroma recombination and omission tests.

### 3.3. Analysis of Metabolic Small Molecules in Chicken Soup

#### 3.3.1. Analysis of the Composition and Differences in Metabolic Small Molecules of Chicken Soup Under Different Treatment Methods

**Figure 5:**
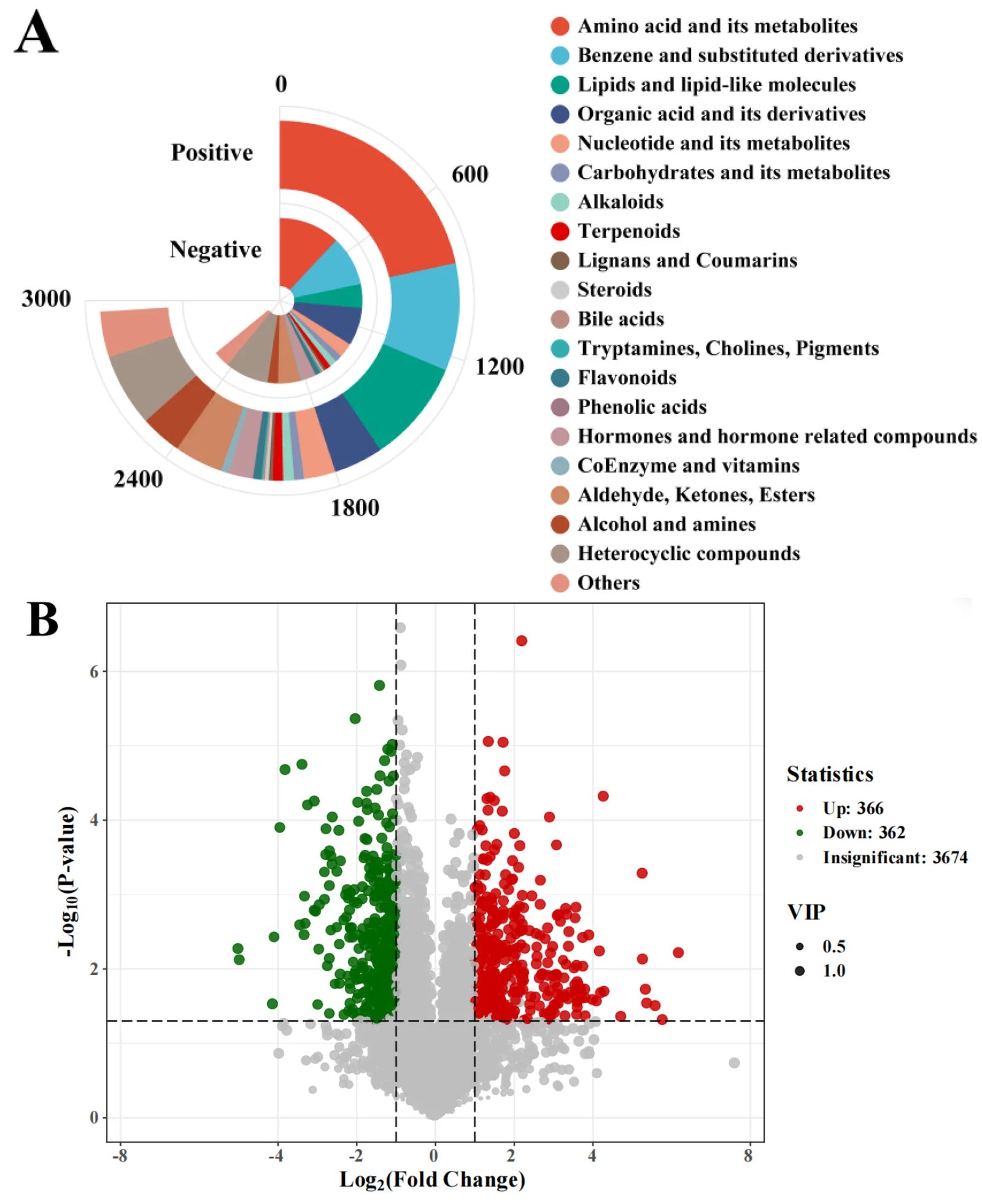
Analysis of the metabolite of chicken soup of different stewing methods: (**A**): The number of metabolite species, (**B**): Volcano plot of differential NVCs. Notes: (**B**): Differential NVCs screened based on VIP > 1, *p* < 0.05, and FC ≥ 2 or ≤0.5.

Figure 5A showed the number of NVCs identified under positive and negative ion modes, which evaluated the differences in metabolite composition between the UAS group and the control group. As shown in Figure 5A, a total of 5032 NVCs (classified into 20 categories) were identified in the chicken soup samples from both groups, with 2676 NVCs detected in positive ion mode and 2356 NVCs detected in negative ion mode. Figure 5A also revealed that the most abundant metabolite categories in both groups of chicken soup were amino acid and its NVCs (24.1%), benzene and substituted derivatives (14.1%), heterocyclic compounds (11.0%), lipids and lipid-like molecules (10.1%), and organic acid and its derivatives (8.7%), etc.

Figure S3A and Figure S3B show the PCA score plots under positive and negative ion modes, respectively, with clear separation between the UAS and control groups. OPLS-DA further confirmed distinct differences in NVC profiles between the two groups, with R^2^X = 0.532, R^2^Y = 0.998, and Q^2^ = 0.936 (Figure S3C). The 1000-permutation test supported the robustness of the model (Figure S3D). The S-plot further identified NVCs with VIP > 1 as the major contributors to group discrimination (Figure S3E).

The volcano plot visually represented the overall distribution characteristics of differential NVCs between the UAS and control groups. In Figure 5B, each point represented an individual metabolite, with the *X*-axis indicating the fold change (log_2_ Fold change) and the *Y*-axis indicating the significance level (−log_10_ *p*-value). The size of each point was proportional to the VIP value from the OPLS-DA model, larger points denoted higher VIP values, indicating greater reliability of the screened differential NVCs. Differential NVCs were screened using the criteria of VIP > 1, *p* < 0.05, and fold change (FC) ≥ 2 or ≤0.5 (Figure 5B). In the plot, red points (log_2_ FC > 1) represented up-regulation differential NVCs, green points (log_2_ FC < −1) represented down-regulation differential NVCs, and gray points represented detected NVCs without significant differences. A total of 728 differential NVCs were identified, including 366 up-regulation and 362 down-regulation NVCs.

Based on the above screening results, key differential NVCs related to flavor were further selected. As presented in Table S2, a total of 322 key differential NVCs were identified, including 166 up-regulation and 166 down-regulation NVCs. These differential NVCs were classified into 7 compound categories, as detailed in Table S2: amino acids and their NVCs (57.2%), lipids and lipid-like molecules (21.7%), benzene and derivatives (4.8%), organic acids and derivatives (3.6%), nucleotides and NVCs (2.4%), carbohydrates and NVCs (1.8%), and other compounds (8.4%). Among these NVCs, amino acids and their NVCs along with lipids and lipid-like molecules emerged as the most abundant differential NVCs. These substances served as both key taste compounds and flavor precursors in chicken soup, also generated diverse VOCs through Maillard reactions, Strecker degradation, lipid oxidation and other processes, collectively constituted the characteristic flavor profile of chicken soup [43].

#### 3.3.2. Analysis of Key Differential Amino Acid and Its NVCs

Amino acids were key taste substances and flavor precursors, with umami, bitter, and sweet amino acids being the main taste-active components [1]. Furthermore, amino acids in the chicken underwent Strecker degradation and the Maillard reaction during the stewing process, produced VOCs including aldehydes, alkanes, alcohols, and aromatic compounds, which collectively formed the complex aroma profiles of the soup [44]. As shown in Figure S4A, a total of 190 differential amino acid and its NVCs were identified, comprising 61 up-regulation and 129 down-regulation NVCs. The most significantly differential NVCs with high VIP value included small peptides such as: Gly-Glu-Gly-Phe-Lys, Leu-Glu-Tyr, Glu-Asp-Met, Glu-Thr-Asp-Arg, Glu-Val-Gln-Glu, Glu-Lys-Leu-Thr-His. Flavor peptides were flavor-bearing protein degradation products in foods, while amino acids served as the fundamental building blocks of proteins, provided insight into the molecular structure and nutritional value of flavor peptides [45]. In this study, the most frequently occurring amino acids in flavor peptides were glutamic acid (Glu), aspartic acid (Asp), alanine (Ala), lysine (Lys), threonine (Thr), and valine (Val). As primary umami-tasting amino acids, Glu and Asp exhibited marked synergistic effects with free amino acids and nucleotides. This combination not only created distinctive soup flavors but also increased the perception of saltiness, serving as a fundamental mechanism behind the unique taste characteristics of umami [45]. Lys and Val primarily imparted bitter tastes, but at higher concentrations, they could enhance flavor complexity and potentiate umami perception [46]. Thr and Ala had sweet taste properties, with these sweet amino acids not only providing a sweetness to chicken soup but also reducing bitterness, thereby improving the overall taste profile [45]. Food proteins were important sources of functional peptides, with small peptides possessing excellent solubility, absorption, and functionality [47]. These amino acids and flavor peptides played crucial roles in protein synthesis, metabolic pathways, and flavor formation [48]. Ultrasonic treatment, through cavitation effects and mechanical action, promoted protein degradation into small peptides and free amino acids, then underwent decarboxylation and deamination reactions to generate compounds such as carbon dioxide, amines and aldehydes, which enriched the flavor complexity of the chicken soup. Cavitation-induced tissue disruption and protein structural changes can facilitate the release of soluble peptides and amino compounds, while these substrates may simultaneously be consumed through Strecker degradation and Maillard reactions [18,47]. Thus, ultrasound may couple precursor release with subsequent chemical transformation into volatile carbonyl and aroma compounds [44].

#### 3.3.3. Analysis of Key Differential Lipids and Lipid-like Compounds

Lipids, as crucial precursors in the formation of chicken flavor, consisted of subcutaneous lipid, intermuscular lipid, and intramuscular lipid, and played an vital role in the nutritional and flavor quality of chicken soup. Among these, intramuscular lipid was the critical component for determining the flavor quality of chicken soup [10]. As shown in Figure S4B, 72 key differential NVCs of lipids and lipid-like compounds were identified between the UAS and the control group, including 60 up-regulation and 12 down-regulation NVCs. These mainly comprised glycerophospholipids (GP), glycerides (GL), and fatty acids (FA). FA, as critical components of lipids, played vital roles in flavor formation in chicken soup. The soup primarily contained oleic acid, linoleic acid, and palmitic acid, which generated various characteristic VOCs through oxidative degradation, such as hexanal, pentanal, 1-octen-3-ol and (E,E)-2,4-decadienal. (E,E)-2,4-decadienal, the primary secondary oxidation product of linoleic acid, as one of the most critical VOCs in chicken soup, was associated with fried and fatty aromas [49]. In this study, 14 free fatty acids (FFA) were identified, with the concentrations being significantly higher (*p* < 0.05) in the UAS group compared to the control group. In chicken soup, the saturated FFA were mainly C14:0, while monounsaturated FFA were mainly represented by palmitoleic acid (C16:1) and oleic acid (C18:1). The polyunsaturated FFA included γ-linolenic acid, eicosapentaenoic acid (EPA, C20:5), and docosapentaenoic acid (DPA, C22:5). Among them, EPA was an important ω-3 fatty acid and was essential for various metabolic processes in the human body and exhibited anti-inflammatory, cardiovascular protective, and immune regulation functions [50]. After ultrasonic treatment, the concentrations of FFA such as palmitoleic acid, oleic acid, and EPA in chicken soup significantly increased (*p* < 0.05). Consequently, both the flavor characteristics and nutritional quality of the chicken soup were enhanced. Additionally, the levels of GP and GL also significantly increased after ultrasonic treatment (*p* < 0.05). This finding confirmed that ultrasonic treatment enhanced the emulsifying properties of chicken soup, mainly due to cavitation effects, which promoted the release of GP and the uniform dispersion of GL, further improved the emulsion stability of the chicken soup. The report of Wu et al. [51] also showed that GP and GL can improve Pickering emulsion stability.

#### 3.3.4. Analysis of Key Differential Organic Acid and Its Derivatives

Organic acids could significantly influence the flavor of meat products [52]. A total of 12 key differential NVCs of organic acid and its derivatives were identified between the UAS group and the control group, including 6 up-regulation and 6 down-regulation NVCs (Figure S4C). These NVCs comprised 4-hydroxybenzoic acid, 2-isopropylmalate, itaconic acid, indolepyruvate, 10-hydroxy-2-decenoic acid, hydrocinnamic acid, caftaric acid and 4-hydroxybenzenesulfonic acid, etc. The concentrations of caftaric acid and 4-hydroxybenzenesulfonic acid in the UAS group were significantly higher than those in the control group (*p* < 0.05), and their acidic properties might have influenced the pH of the chicken soup. Moreover, 4-hydroxybenzoic acid, a phenolic derivative of benzoic acid, exhibited antioxidant, antibacterial, and anti-inflammatory properties, which positively affected cardiovascular health. Moreover, itaconic acid, hydrocinnamic acid, and similar compounds demonstrated potent antioxidant activity, efficiently neutralizing free radicals formed during the stewing process [53,54]. These compounds inhibited hyaluronic acid formation, reduced heterocyclic amine production, and delayed lipid and protein oxidation processes. Indolepyruvate and 2-isopropylmalate were primarily involved in the biosynthesis and metabolic pathways of tryptophan and leucine [13], respectively, with tryptophan being a sweet-tasting amino acid and leucine being an essential amino acid. Figure S4C showed that the UAS group exhibited significantly higher levels of these two key NVCs than the control group. Therefore, ultrasonic treatment improved the physicochemical properties, functional characteristics, and flavor properties of chicken soup by enriching organic acid and its derivatives.

#### 3.3.5. Analysis of Key Differential Nucleotide and Its NVCs

Nucleotide and its NVCs were critical flavor enhancers in chicken products, exhibiting synergistic effects with umami substances such as Glu to enhance the characteristic umami taste [55]. A total of 8 key differential nucleotide and its NVCs were identified in the chicken soup from the UAS group and the control group, including 6 up-regulation and 2 down-regulation NVCs (Figure S4D). These NVCs included 2′-deoxyadenosine-5′-monophosphate, xanthosine, xanthine, 2′-deoxyinosine and cyclic guanosine monophosphate, etc. The concentrations of 2′-deoxyinosine, xanthine, and xanthosine in the UAS group were significantly higher than those in the control group (*p* < 0.05). Notably, 2′-deoxyinosine was derived from the 5′-inosine monophosphate (IMP), as a flavor nucleotide, which was primarily formed by the degradation of adenosine triphosphate (ATP) in muscle tissue. IMP exhibited a strong umami and sweet taste and could synergize with 5′-adenylic acid to enhance umami and suppress bitterness [56]. Furthermore, xanthine was a secondary metabolite of ATP, generated by the dephosphorylation of 5′-xanthosine monophosphate (5′-XMP) [53]. Fu et al. [57] reported that 5′-XMP played a crucial role in umami. Therefore, ultrasonic treatment facilitated the release of nucleotide and its NVCs and further promoted the umami of the chicken soup.

#### 3.3.6. Analysis of Key Differential Carbohydrates and Its NVCs

The primary polysaccharides in animal muscle tissue consisted of glycogen, proteoglycans, and glycoproteins. Among these, glycogen served as an essential energy reserve and played a crucial role during muscle activity, while glycoproteins and inosine monophosphate contributed to the formation of characteristic umami compounds during heat treatment [58]. A total of 6 key differential carbohydrates and its NVCs were detected in the chicken soup from both the UAS and control groups, including 5 up-regulation and 1 down-regulation NVCs (Figure S4E). These NVCs consisted of cellobiose, 1-kestose, maltopentaose, D-fructose-6-phosphate-disodium salt, melezitose and cellotetraose. 1-kestose was a trisaccharide composed of two fructose units and one glucose unit linked by β (2→1) glycosidic bonds, classified as a natural sweetener [59]. As shown in Figure S4E, the 1-kestose concentration in the UAS group was significantly higher than that in the control group (*p* < 0.05), consistent with the result of total sugar concentration [8]. Ultrasonic treatment promoted the dissolution of carbohydrates and thereby increased the total sugar concentration. Moreover, NVCs such as cellobiose, maltopentaose, melezitose, cellotetraose could be hydrolyzed into reducing sugars like glucose. During the stewing process, reducing sugars reacted with amino acids through Maillard reaction, then characteristic flavor substances were formed [9].

#### 3.3.7. Analysis of Key Differential Benzene and Substituted Derivatives

Benzene and substituted derivatives primarily contributed to the aroma enhancement in chicken soup. As shown in Figure S4F, a total of 16 key differential benzene and substituted derivatives were identified in the chicken soup from both the UAS and control groups, including 11 up-regulation and 5 down-regulation NVCs. These included hexylresorcinol, shogaol, [10]-shogaol, 2,5-dihydroxybenzoic acid, 4-(methylthio) benzoic acid, (10)-gingerol, (8)-gingerol and 2,4,6-trihydroxybenzoic acid, etc. The concentrations of shogaol, [10]-shogaol, (10)-gingerol, (8)-gingerol in the UAS group were significantly higher than those in the control group (*p* < 0.05). These compounds were derived from the ginger, indicating that ultrasonic treatment enhanced the contribution of ginger to the chicken soup. Furthermore, the control group exhibited significantly higher levels of 2,5-dihydroxybenzoic acid and 2,4,6-trihydroxybenzoic acid than the UAS group (*p* < 0.05). These key differential NVCs served as precursor of benzoic aldehyde, which was the key VOCs. Phenylalanine, an aromatic free amino acid, reacted with reducing sugars though Maillard reaction, leading to the formation of benzoic aldehyde and other volatile small molecules compounds [24]. The cavitation effect generated by ultrasonic treatment created high-temperature and high-pressure conditions, promoted the decarboxylation of benzoic acid derivatives, then resulted in the production of benzoic aldehyde. The UAS group showed a significant decrease concentration of benzoic acid derivatives (*p* < 0.05) and a significant increase level of benzoic aldehyde (*p* < 0.05), demonstrating that ultrasonic treatment facilitated the formation of key aromatic compounds (especially benzoic aldehyde) in chicken soup.

#### 3.3.8. Analysis of Other Key Differential NVCs

Furthermore, 28 other key differential NVCs were identified (Figure S4G), mainly including flavonoids, coenzymes and vitamins, terpenoids, phenolic acids, as well as aldehydes, ketones, esters, alcohols, and amines. The flavonoids detected in both the UAS and control groups were up-regulation NVCs. Among flavonoids, compounds such as phloretin, cynarine, naringin possessed biological activity including antioxidant, anti-inflammatory, and antidiabetic effects [60]. Phloretin and cynarine exhibited multiple pharmacological activities, such as antioxidant, hypoglycemic, anti-inflammatory, and antitumor effects [61,62]. Naringin, a natural flavonoid compound, imparted a bitter taste and also demonstrated antioxidant, anti-inflammatory, and antimicrobial properties [63]. These findings suggested that chicken soup, enriched in flavonoids, might exert positive health benefits. The coenzymes and vitamins in chicken soup mainly included biotin and vitamin K. Biotin (Vitamin B7), a water-soluble vitamin, was an essential nutrient for maintaining metabolic balance and promoting overall health [64]. Vitamin K was a trace nutrient critical for blood coagulation and bone health, and might play a role in lowering the risk of cardiovascular diseases [65]. The concentrations of vitamins in the UAS group were significantly higher than those in the control group (*p* < 0.05), which due to the enhanced release of vitamins by ultrasonic treatment. The terpenoids and phenolic acids identified included capsianoside I, capsianoside IV, picrasin F, nigakilactone I and methylgingerol. Among these, 2 NVCs were up-regulation and 3 down-regulation, picrasin F and nigakilactone I, known as bitter substances, had lower levels in the UAS group than in the control soup. This demonstrated that ultrasonic treatment promoted their degradation, thereby reduced the bitterness of the chicken soup.

### 3.4. Potential Pathways for the Generation of Volatile Organic Compounds

**Figure 6:**
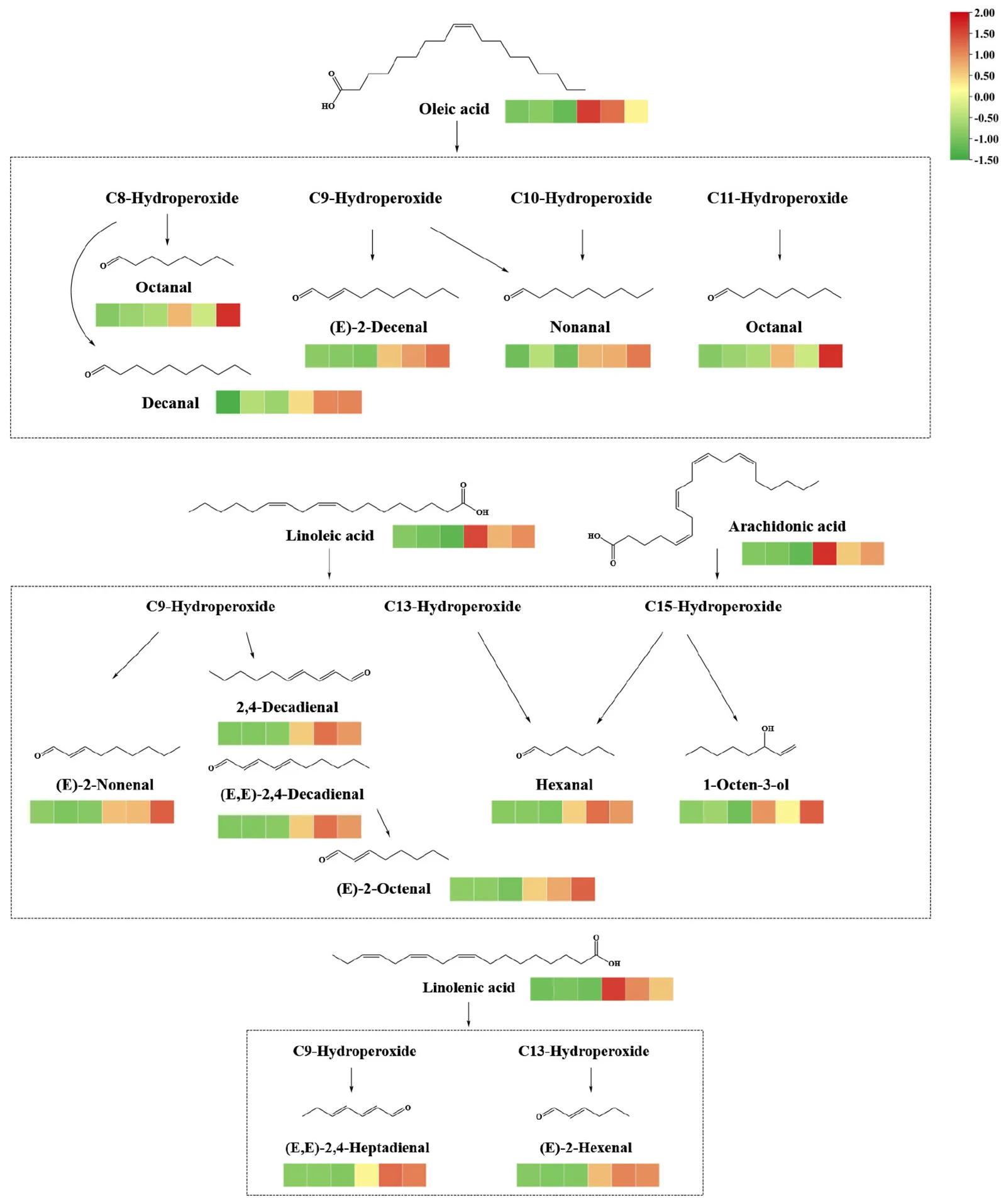
The proposed degradation pathways of oleic acid, linoleic acid, arachidonic acid, and linolenic acid, respectively. Colors indicate the relative levels of the compounds (green = low, yellow = moderate, red = high). For each compound, the six horizontal colored blocks from left to right represent Control 1, Control 2, Control 3, UAS 1, UAS 2, and UAS 3, respectively.

The unique flavor of chicken soup primarily resulted from complex chemical reactions occurring during the stewing process. During stewing, lipids underwent hydrolysis and oxidation, generating flavor precursors that enriched the diversity of VOCs. As shown in Figure 6, Unsaturated fatty acids were more susceptible to oxidation than saturated fatty acids, leading to the generation of VOCs through a series of reactions. Furthermore, oleic acid, linoleic acid, linolenic acid and arachidonic acid contained one, two, three and four double bonds, respectively, and were the primary contributors to the oxidation of unsaturated fatty acids (UFAs) in meat [66]. The generation of alcohols was primarily attributed to the oxidation of UFAs accompanied and the positional isomerization of double bonds along the carbon chain. The direct catabolism of arachidonic acid, a fatty acid with four double bonds, leads to the formation of 1-octen-3-ol [67]. During lipid oxidation, unsaturated fatty acids form various hydroperoxides, which were then converted into aldehydes via carbon-carbon bond cleavage catalyzed by hydroperoxide cleavage enzymes [22]. Oleic acid underwent oxidation to generate C8, C9, C10, and C11 hydroperoxides. The C8 and C11 hydroperoxides underwent homolytic cleavage to form octanal, while the C9 hydroperoxide yielded (E)-2-decenal, and the C10 hydroperoxide yielded nonanal. Furthermore, the keto-enol tautomerism of C8 and C9 hydroperoxides may lead to the formation of decanal and nonanal [66]. Distinct hydroperoxides were also produced from linoleic acid and arachidonic acid via homolytic cleavage. Linoleic acid oxidation generated C9 and C13 hydroperoxides. The C13 hydroperoxide underwent β-scission to form hexanal (a product also generated from C15 hydroperoxide cleavage in arachidonic acid oxidation). The C9 hydroperoxide primarily yielded (E,E)-2,4-decadienal and (E,Z)-2,4-decadienal, with minor amounts of (E)-2-nonenal and (E)-2-octenal as secondary cleavage products [23]. The oxidation of linolenic acid produced C9 and C13 hydroperoxides. C13 hydroperoxides mainly led to the formation of trans-2-hexenal, while (E,E)-2,4-heptadienal was primarily generated from C9 hydroperoxides or secondary oxidation [68]. In addition, the Maillard reaction was one of the key pathways for the formation of characteristic flavors and color during thermal processing of chicken soup. Ultrasound treatment, through cavitation, thermal, and mechanical effects, disrupted the tissue structure of chicken, thereby accelerating the release of flavor precursors. This promoted key flavor-forming reactions such as lipid oxidation and the Maillard reaction, thereby increasing the efficiency of VOCs formation and contributing to a richer and more balanced aroma in chicken soup.

## 4. Conclusions

This study used GC-MS and UPLC-MS/MS to elucidate the regulatory effects of UAS on the flavor profile and metabolite composition of chicken soup. A total of 39 VOCs were identified in terms of flavor components. Among them, 7 VOCs, including (E,E)-2,4-decadienal, (E)-2-octenal, and (E,E)-2,4-hexadienal, were confirmed as key characteristic VOCs through aroma recombination and omission tests. UAS significantly enhanced the concentrations of these VOCs and optimized their overall aroma contributions to the volatile profile. In addition, untargeted metabolomics analysis detected a total of 5032 NVCs classified into 20 categories, including a high proportion of amino acid and its NVCs, lipids and lipid-like compounds and benzene and substituted derivatives. Among these NVCs, 322 were identified as key differential NVCs, exhibiting higher abundances in the UAS group. In particular, significant increases were observed in flavor precursors including small peptides, fatty acids, and nucleotides. In conclusion, UAS enhanced the flavor complexity of chicken soup by facilitating the release of flavor precursors, including small peptides, nucleotides, and lipid-derived NVCs, and by promoting the formation of key VOCs. This study provided a methodological basis for investigating and modulating flavor formation in food processing.

## Figures and Tables

**Table 1:** Odor activity values (OAVs) of volatile flavor compounds in chicken soup with different treatment method.

Code	Compounds	CAS	Threshold (in Water, mg/kg)	0	C30	C60	C90	C120	U30	U60	U90	U120
1	Pentanal	110-62-3	0.012	-	-	-	-	-	-	19.40	22.70	30.70
2	Hexanal	66-25-1	0.0045	58.93	59.76	97.55	99.82	107.50	239.35	459.91	495.67	473.62
3	(E)-2-hexenal	6728-26-3	0.11	-	1.94	2.91	3.32	3.49	28.52	41.45	55.72	49.45
4	Heptanal	111-71-7	0.0028	99.92	211.59	217.97	226.47	235.80	295.31	992.22	1143.90	1226.21
5	(E,E)-2,4-hexadienal	142-83-6	0.06	-	-	-	-	-	8.54	9.70	11.28	9.22
6	(E)-2-heptenal	18829-55-5	0.013	15.05	16.25	18.51	38.33	43.67	78.38	128.20	179.06	147.52
7	Benzoic aldehyde	100-52-7	0.35	1.21	1.25	1.48	1.58	1.94	2.95	3.22	4.83	3.86
8	Octanal	124-13-0	0.0007	122.90	143.92	233.39	351.83	392.08	156.23	538.36	892.34	805.15
9	(E,E)-2,4-heptadienal	4313-03-5	0.056	6.78	8.11	9.41	15.98	24.41	22.90	60.62	61.39	61.73
10	(E)-2-octenal	2548-87-0	0.003	463.22	524.38	689.38	715.95	817.98	567.95	781.59	961.79	1246.28
11	Nonanal	124-19-6	0.001	1738.64	1848.74	2007.57	2212.84	2467.34	4092.24	5971.67	6066.21	8515.52
12	(E)-2-nonenal	18829-56-6	0.00008	2798.58	5220.51	7159.67	8934.67	11,719.94	21,139.47	82,006.98	107,634.59	95,474.12
13	Decanal	112-31-2	0.0001	-	1453.76	1699.38	2844.12	3954.80	751.54	4191.23	4619.24	6452.95
14	(E,E)-2,4-nonadienal	5910-87-2	0.00009	-	-	-	-	-	-	6824.99	10,944.36	11,822.14
15	(Z)-3,7-dimethylocta-2,6-dienal	106-26-3	0.03	-	-	-	2.31	-	51.22	80.72	139.39	91.70
16	(E)-2-decenal	3913-81-3	0.0003	529.87	600.83	667.14	735.59	807.34	931.23	940.17	1445.92	1680.40
17	Citral	5392-40-5	0.1	-	2.22	2.60	2.87	3.06	4.13	4.92	5.77	5.80
18	(E,Z)-2.4-decadienal	25152-83-4	0.0003	-	722.91	1036.94	1198.73	1253.27	2314.14	3157.94	4284.43	7701.33
19	(E,E)-2,4-decadienal	25152-84-5	0.00007	-	-	-	-	-	1830.51	5066.42	8613.43	7277.74
20	(E,E)-2,4-undecanal	30361-29-6	0.001	-	432.66	445.29	485.00	514.37	1061.84	1550.57	2264.58	4087.64
21	Tetradecanal	124-25-4	0.064	1.69	2.14	5.14	5.97	6.41	2.26	5.33	6.73	10.46
22	1-Octen-3-ol	3391-86-4	0.001	1075.91	1185.19	1229.08	2137.26	3075.46	5613.77	15,550.61	16,607.31	15,057.51
23	Borneol	507-70-0	0.14	4.15	4.66	5.51	13.06	13.50	60.84	244.26	307.73	278.10
24	Alpha-terpineol	98-55-5	0.33	1.42	2.52	2.74	2.95	3.13	3.70	7.61	9.83	12.18
25	6-Methyl-5-hepten-2-one	110-93-0	0.05	8.87	11.04	12.90	17.25	33.43	50.51	101.35	129.23	122.06
26	2-Pentylfuran	3777-69-3	0.006	105.71	112.73	121.88	196.98	215.81	198.35	271.12	351.11	463.87

Note: Odor thresholds in water were obtained from reported literature values; “-” indicates not detected.

**Table 2:** Influence of key differential aroma active compounds on the overall aroma profiles.

Compound(s) Omitted	n/N	Significance	Aroma Description	Chemical Structure
(E)-2-hexenal	7	*	Pungent, marzipan	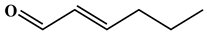
(E,E)-2,4-hexadienal	10	***	Sweet, spicy	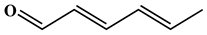
(E)-2-heptenal	7	*	Fatty, spicy, fruity	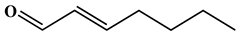
Benzoic aldehyde	4	-	Almond-like, nutty	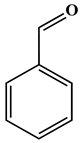
1-Octen-3-ol	6	-	Mushroom	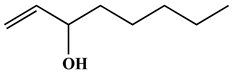
(E,E)-2,4-heptadienal	8	**	Chickeny, fatty	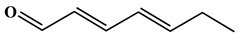
(E)-2-octenal	10	***	Fruity, fatty	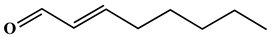
(E,Z)-2,4-decadienal	7	*	Fatty, tallowy	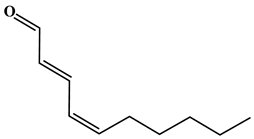
(E,E)-2,4-decadienal	10	***	Fatty, chickeny	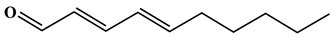
(E,E)-2,4-undecanal	6	-	Meaty, tallowy	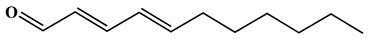

Note: n: Number of panelists perceived differences in the triangle test correctly; N: number of panelists participate in the triangulation test. Significance: ***, *p* < 0.001, extremely significant; **, *p* ≤ 0.01, highly significant; *, *p* ≤ 0.05, significant; -: no significant.

## Data Availability

The original contributions presented in the study are included in the article and Supplementary Materials, further inquiries can be directed to the corresponding authors.

## Supplementary Materials

The following supporting information can be downloaded at: https://www.mdpi.com/article/10.3390/foods15172978/s1, Table S1: The VOCs and their concentration identified by SPME GC-MS method in chicken soup. Table S2: Proportions and types of key differential non-volatile compounds (NVCs) of chicken soup of different stewing methods. Figure S1: Analysis of the volatile organic compounds of chicken soup of different treatment method: A: PCA plot, B: OPLS-DA plot, C: Permutation test based on OPLS-DA analysis, D: VIP scores plots based on OPLS-DA analysis. Notes: C: Cross-validation results obtained after 1000 permutation tests. D: Change patterns of volatile flavor compounds with VIP >1 based on OPLS-DA analysis (C = Control; U = UAS). Figure S2: Screening of key differential volatile organic compounds in chicken soup under different stewing methods. Figure S3: Analysis of the metabolite of chicken soup of different stewing methods: A: PCA plot under positive ion pattern, B: PCA plot under negative ion pattern, C: OPLS-DA plot, D: Permutation test based on OPLS-DA analysis, E: S-plot based on OPLS-DA analysis. Notes: D: Cross-validation results obtained after 1000 permutation tests. E: Change patterns of NVCs with VIP >1 (red) and ≤1 (green) based on OPLS-DA analysis. Figure S4: Analysis of the key differential NVCs: A: Amino acids and derivatives, B: Lipids and lipid-like compounds, C: Organic acid and its derivatives, D: Nucleotides and derivatives, E: Carbohydrates and derivatives, F: Benzene and substituted derivatives, G: Others. Notes: Different colors represent different concentrations. (green = low, yellow = moderate, red = high).

## Abbreviations

The following abbreviations are used in this manuscript:

UAS	ultrasonic-assisted stewing
VOCs	volatile organic compounds
NVCs	non-volatile compounds
GC-MS	gas chromatography-mass spectrometry
SPME	solid-phase microextraction
UPLC-MS/MS	ultra-performance liquid chromatography-tandem mass spectrometry
LC-MS/MS	liquid chromatography-tandem mass spectrometry
OAV	odor activity value
MNPs	micro-nano particles
PTFE	polytetrafluoroethylene
PDMS/DVB	polydimethylsiloxane/divinylbenzene
EI	electron impact
NIST	National Institute of Standards and Technology
RI	retention index
QC	quality control
TIC	total ion chromatogram
SVR	support vector regression
HMDB	Human Metabolome Database
KEGG	Kyoto Encyclopedia of Genes and Genomes
SD	standard deviation
ANOVA	analysis of variance
LSD	least significant difference
PCA	principal component analysis
OPLS-DA	orthogonal partial least squares discriminant analysis
VIP	variable importance in projection
FC	fold change
GP	glycerophospholipids
GL	glycerides
FA	fatty acids
FFA	free fatty acids
EPA	eicosapentaenoic acid
DPA	docosapentaenoic acid
IMP	inosine monophosphate
ATP	adenosine triphosphate
XMP	xanthosine monophosphate
UFA	unsaturated fatty acid
