# Supplementary material for: Ultrasonic-Assisted Stewing Enhances Chicken Soup Flavor by Promoting Flavor Precursor Release and Key Aroma Compound Formation
Source: Foods. 2026 Aug 25;15(17):2978. doi: 10.3390/foods15172978 (PMC13565453; doi:10.3390/foods15172978)
Supplement: Supplementary file 1 [file foods-15-02978-s001.zip › foods-4487527-supplementary.pdf]

Supplementary material

Ultrasonic-Assisted Stewing Enhances Chicken Soup Flavor by Promoting Flavor Precursor Release and Key Aroma Compound Formation

Dandan Zhang[a], Ziyan Yue[b, c],Ruiying Chen[b], Huanjuan Guo[b], Weikang Guo[b], Cuiping Feng[b], Yingchun Zhu[b,*]

[a]Shanxi Agricultural University, Taigu 030801, China

[b]College of Food Science and Engineering, Shanxi Agricultural University, Taigu 030801, China

[c]School of Food and Health, Beijing Technology and Business University, Beijing, 100048, China

zhangdandan@sxau.edu.cn (D.Z.); 15525794820@163.com (Z.Y.); 18734422667@163.com (R.C.); 13485436066@163.com (H.G.); 17835265718@163.com (W.G.); ndfcp@163.com (C.F.)

* Correspondence: yingchun0417@163.com; Tel.:+86-185-0344-9054

Table S1 The VOCs and their concentration identified by SPME GC-MS method in chicken soup

Code	Compounds	CAS	Identification	Concentration (mg/kg)									RI
				0	C30	C60	C90	C120	U30	U60	U90	U120	
A1	Pentanal	110-62-3	MS	-	-	-	-	-	-	0.23±0.05a	0.27±0.04a	0.37±0.20a	-
A2	(E)-2-pentenal	1576-87-0	MS	-	-	-	-	-	0.41±0.01b	0.44±0.21b	0.58±0.20b	0.95±0.13a	-
A3	Hexanal	66-25-1	MS	0.27±0.10c	0.27±0.04c	0.44±0.25bc	0.45±0.37bc	0.48±0.04bc	1.08±0.26b	2.07±0.35a	2.23±0.10a	2.13±0.34a	-
A4	(E)-2-hexenal	6728-26-3	MS, RI	-	0.21±0.06d	0.32±0.12d	0.37±0.05d	0.38±0.07d	3.14±0.31c	4.56±0.32b	6.13±0.81a	5.44±0.61ab	870
A5	Heptanal	111-71-7	MS, RI	0.28±0.14b	0.59±0.12b	0.61±0.01b	0.63±0.31b	0.66±0.02b	0.83±0.06b	2.78±0.53a	3.20±0.64a	3.43±0.41a	917
A6	(E,E)-2,4-hexadienal	142-83-6	MS, RI	-	-	-	-	-	0.51±0.05a	0.58±0.12a	0.68±0.03a	0.55±0.15a	934
A7	(E)-2-heptenal	18829-55-5	MS, RI	1.00±0.59c	1.51±0.04c	1.61±0.03c	1.68±0.08c	1.76±0.10c	9.81±0.86b	11.62±0.56ab	12.85±0.19a	12.97±2.07a	969
A8	Benzoic aldehyde	100-52-7	MS, RI	0.20±0.05d	0.21±0.07d	0.24±0.11d	0.50±0.02d	0.57±0.14d	1.02±0.20c	1.67±0.26b	2.33±0.13a	1.92±0.17ab	976
A9	Octanal	124-13-0	MS, RI	0.42±0.02e	0.44±0.09de	0.52±0.13de	0.55±0.17cde	0.68±0.08cde	1.03±0.04bcd	1.13±0.12abc	1.69±0.31a	1.35±0.47ab	1006
A10	(E,E)-2,4-heptadienal	4313-03-5	MS, RI	1.08±0.11c	1.19±0.02c	1.23±0.55c	2.14±0.31bc	3.08±0.29bc	5.61±0.30b	15.55±1.35a	16.61±0.95a	15.06±3.79a	1020
A11	(E)-2-octenal	2548-87-0	MS, RI	0.44±0.11e	0.55±0.01de	0.64±0.07de	0.86±0.06de	1.67±0.26cd	2.53±0.03c	5.07±0.61b	6.46±0.04a	6.10±1.01ab	1072
A12	Nonanal	124-19-6	MS, RI	0.63±0.03d	0.68±0.01d	0.73±0.23d	1.18±0.67cd	1.29±0.31cd	1.19±0.13bcd	1.63±0.12bc	2.11±0.23ab	2.78±0.20a	1121
A13	(E)-2-nonenal	18829-56-6	MS, RI	0.51±0.05d	0.53±0.07d	0.57±0.09cd	0.68±0.27cd	0.74±0.06cd	1.12±0.10c	1.77±0.04b	1.84±0.33ab	2.39±0.0.38a	1170
A14	Decanal	112-31-2	MS, RI	0.09±0.01c	0.10±0.01c	0.16±0.05c	0.25±0.11bc	0.27±0.08bc	0.11±0.02bc	0.38±0.05ab	0.62±0.22a	0.56±0.06a	1210
A15	(E,E)-2,4-nonadienal	5910-87-2	MS, RI	0.38±0.19c	0.45±0.02c	0.53±0.14c	0.89±0.16bc	1.37±0.17b	1.28±0.08b	3.39±0.25a	3.44±0.25a	3.46±0.56a	1224
A16	(Z)-3,7-dimethylocta-2,6-dienal	106-26-3	MS, RI	1.39±0.61c	1.57±0.07bc	2.07±0.44bc	2.15±0.31bc	2.45±0.14bc	1.70±0.06bc	2.34±0.21abc	2.89±0.63ab	3.74±0.99a	1248
A17	(E)-2-decenal	3913-81-3	MS, RI	0.65±0.24d	0.68±0.03d	0.71±0.25d	1.41±0.47cd	2.11±0.14cd	1.91±0.25c	3.88±0.55b	4.58±0.86ab	5.84±0.64a	1275
A18	Citral	5392-40-5	MS, RI	1.74±0.13d	1.85±0.39d	2.01±0.04cd	2.21±0.45cd	2.47±0.12cd	4.09±0.75bc	5.97±0.60b	6.07±0.63b	8.52±1.80a	1282
A19	(E,Z)-2.4-decadienal	25152-83-4	MS, RI	0.22±0.20c	0.42±0.04c	0.57±0.04c	0.71±0.08c	0.94±0.12c	1.69±0.29c	6.56±0.66b	8.61±0.71a	7.64±1.25ab	1310
A20	(E,E)-2,4-decadienal	25152-84-5	MS, RI	0.58±0.59d	0.65±0.01d	0.77±0.12d	1.83±0.28d	1.89±0.19d	8.52±0.24c	34.20±4.44b	43.08±1.63a	38.93±1.65ab	1334
A21	2-Undecenal	2463-77-6	MS, RI	0.47±0.17e	0.83±0.06de	0.91±0.03de	0.97±0.16de	1.03±0.14de	1.22±0.13d	2.51±0.31c	3.24±0.28b	4.02±0.44a	1374

A22	(E,E)-2,4-undecanal	30361-29-6	MS, RI	-	0.20±0.01bc	0.23±0.02bc	0.25±0.06bc	0.28±0.06bc	0.75±0.05b	1.78±0.42a	1.82±0.28a	1.73±0.38a	1389
A23	Tetradecanal	124-25-4	MS, RI	-	0.15±0.07de	0.17±0.05de	0.28±0.02cde	0.40±0.14bcd	0.08±0.03bc	0.42±0.02ab	0.46±0.16ab	0.65±0.10a	1603
	Aldehyde			10.34	13.08	15.04	20.00	24.52	49.62	110.52	131.80	130.53	
B1	Camphene	79-92-5	MS, RI	-	-	-	-	-	-	0.61±0.04b	0.98±0.17a	1.06±0.27a	958
B2	2,4-Octadien	13643-08-8	MS	-	-	-	0.07±0.00d	-	1.54±0.20c	2.42±0.02b	4.18±0.20a	2.75±0.41b	-
B3	3,7-Dimethyl-1-octene	4984-01-4	MS	0.16±0.02a	0.18±0.02a	0.20±0.02a	0.22±0.02a	0.24±0.00a	0.28±0.14a	0.28±0.13a	0.43±0.24a	0.50±0.20a	-
B4	Pentadecan	629-59-4	MS, RI	-	0.22±0.03c	0.26±0.04c	0.29±0.05bc	0.31±0.02bc	0.41±0.01abc	0.49±0.11ab	0.58±0.11a	0.58±0.14a	1509
B5	Hexadecane	629-62-9	MS, RI	-	0.22±0.11e	0.31±0.22de	0.36±0.08de	0.38±0.06de	0.69±0.01cd	0.95±0.012bc	1.29±0.19b	2.31±0.34a	1597
B6	2,6,8-Trimethyldecane	62108-26-3	MS	-	-	-	-	-	0.13±0.03bc	0.35±0.05abc	0.60±0.41a	0.51±0.06ab	-
B7	9-Hexyl-heptadecane	55124-79-3	MS	0.13±0.07a	0.14±0.01a	0.14±0.06a	0.14±0.01a	0.15±0.00a	0.23±0.08a	0.26±0.09a	0.28±0.09a	0.31±0.12a	-
B8	Heptadecane	629-78-7	MS, RI	-	0.43±0.17de	0.45±0.04de	0.49±0.05de	0.51±0.01de	1.06±0.38cd	1.55±0.26c	2.26±0.24b	4.09±0.44a	1703
B9	Octadecane	629-92-5	MS, RI	-	0.39±0.09cd	0.47±0.09cd	0.49±0.09bcd	0.52±0.01bcd	0.52±0.03bcd	0.55±0.09bc	1.01±0.21b	2.23±0.48a	1796
	Hydrocarbon			0.29	1.58	1.83	2.05	2.11	4.86	7.47	11.62	14.34	
C1	1-Octen-3-ol	3391-86-4	MS, RI	0.04±0.02d	0.05±0.01d	0.07±0.02d	0.07±0.01d	0.15±0.06cd	0.32±0.02c	0.36±0.06bc	0.45±0.14ab	0.63±0.16a	986
C2	Borneol	507-70-0	MS, RI	0.12±0.01b	0.15±0.02b	0.17±0.03b	0.17±0.11b	0.20±0.02b	0.16±0.02b	0.23±0.10b	0.26±0.11b	0.49±0.11a	1183
C3	Alpha-terpineol	98-55-5	MS, RI	0.34±0.03d	0.46±0.12cd	0.72±0.53bcd	0.82±0.02bcd	1.08±0.19bcd	0.61±0.20bc	1.17±0.07ab	1.81±0.36a	1.22±0.12ab	1198
C4	2-Decyn-1-ol	4117-14-0	MS, RI	-	-	-	0.18±0.10bc	0.20±0.11b	0.18±0.05b	0.21±0.09b	0.39±0.06a	0.43±0.01a	1199
C5	2,6-Octadiene-4,5-diol	4486-59-3	MS	-	-	-	-	-	-	-	-	0.78±0.07a	-
	Alcohol			0.50	0.66	0.97	1.24	1.64	1.27	1.97	2.92	3.55	
D1	6-Methyl-5-hepten-2-one	110-93-0	MS, RI	-	0.11±0.11e	0.37±0.10de	0.50±0.18cde	0.73±0.11bcd	0.29±0.09bcd	0.41±0.13abc	0.66±0.17ab	0.99±0.24a	991
D2	2-Pentylfuran	3777-69-3	MS, RI	0.11±0.01c	0.14±0.03c	0.33±0.03c	0.38±0.00b	0.41±0.02b	0.14±0.01b	0.34±0.05b	0.43±0.09b	0.67±0.03a	993
	Other			0.11	0.25	0.70	0.88	1.14	0.43	0.75	1.09	1.66	
	Total concentration			11.24	15.57	18.53	24.17	29.41	56.19	120.70	147.43	150.08	

Note: Different lowercase letters indicate significant differences among values in the same row ($P < 0.05$); A: aldehydes; B: hydrocarbons; C: alcohols; D: others; ‘-’ indicates not detected or RI value not calculable. RI reference values were obtained from the NIST Chemistry WebBook (http://webbook.nist.gov/chemistry/).

Table S2 Proportions and types of key differential non-volatile compounds (NVCs) of chicken soup of different stewing methods

Classification	Proportion	NVCs with higher abundance	NVCs with lower abundance
Amino acids and derivatives	57.2%	61	129
Lipids and lipid-like molecules	21.7%	62	10
Benzene and substituted derivatives	4.8%	11	5
Organic acid and its derivatives	3.6%	6	6
Nucleotides and derivatives	2.4%	6	2
Carbohydrates and derivatives	1.8%	5	1
Others	8.4%	15	13

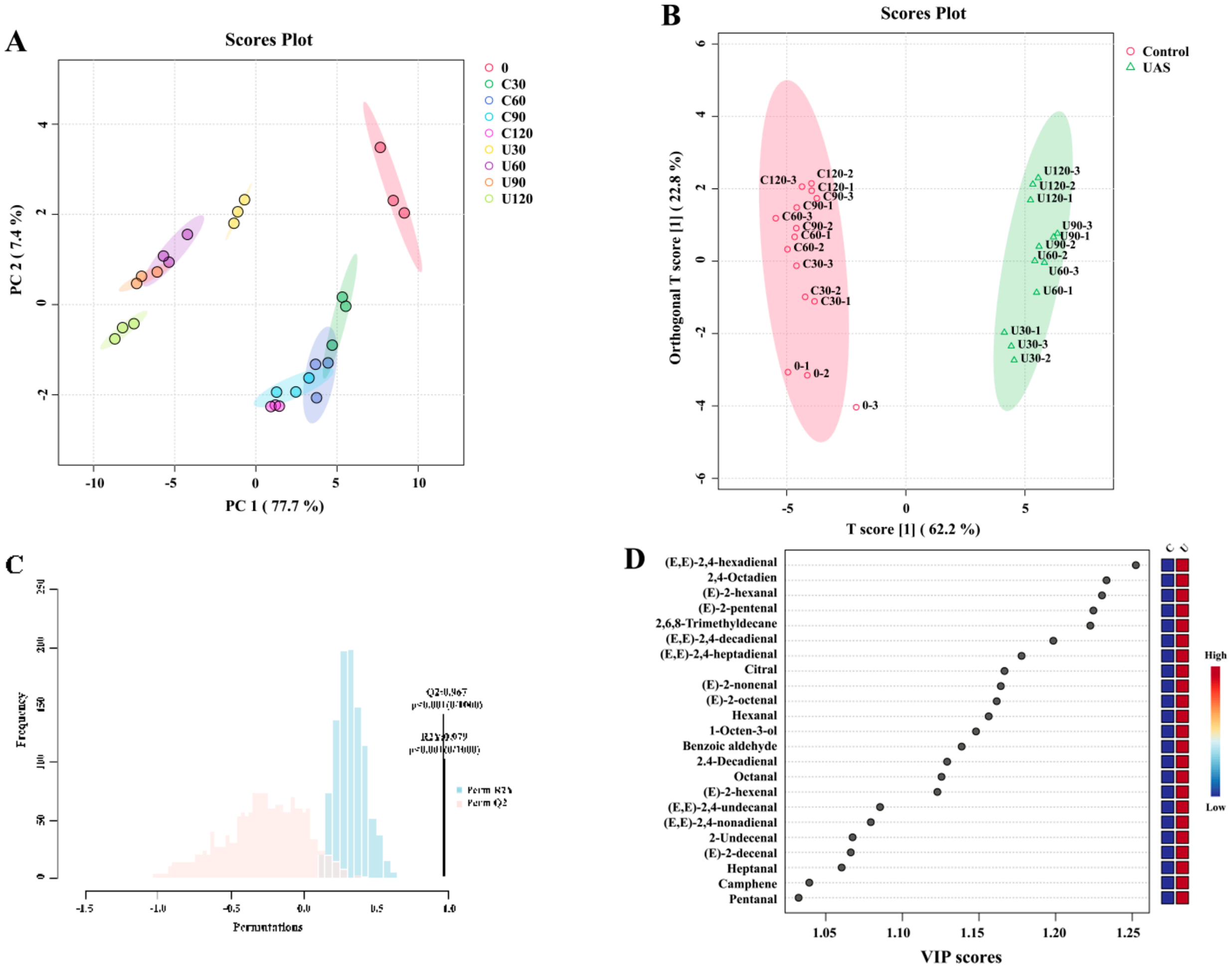

Figure S1. Analysis of the volatile organic compounds of chicken soup of different treatment method: A: PCA plot, B: OPLS-DA plot, C: Permutation test based on OPLS-DA analysis, D: VIP scores plots based on OPLS-DA analysis. Notes: C: Cross-validation results obtained after 1000 permutation tests. D: Change patterns of volatile flavor compounds with VIP >1 based on OPLS-DA analysis (C = Control; U = UAS).

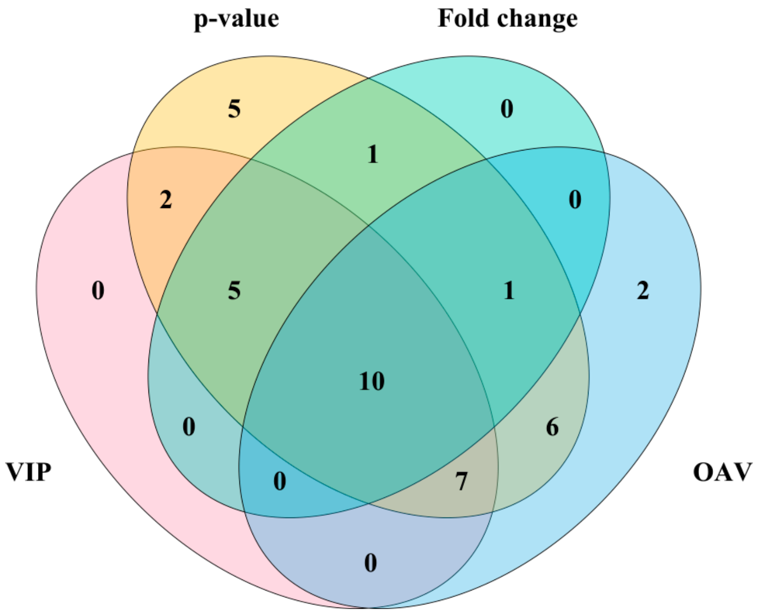

Figure S2. Screening of key differential volatile organic compounds in chicken soup under different stewing methods.

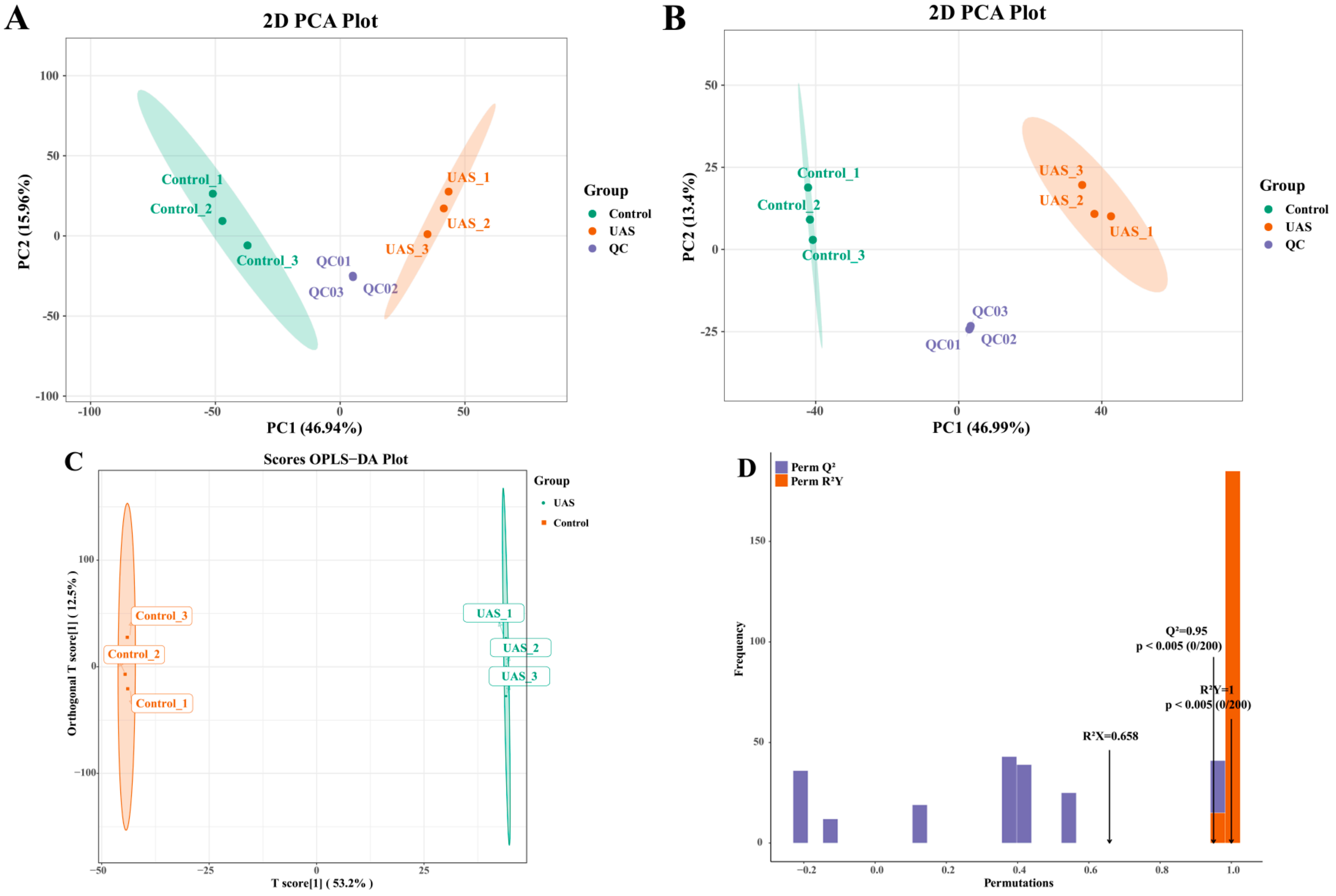
A
2D PCA Plot
PC2 (15.96%)
PC1 (46.94%)
Control_1
Control_2
Control_3
UAS_1
UAS_2
UAS_3
QC01
QC02
QC03
Group
Control
UAS
QC
B
2D PCA Plot
PC2 (13.4%)
PC1 (46.99%)
Control_1
Control_2
Control_3
UAS_1
UAS_2
UAS_3
QC01
QC02
QC03
Group
Control
UAS
QC
C
Scores OPLS−DA Plot
Orthogonal T score[1] (12.5%)
T score[1] (53.2%)
Control_1
Control_2
Control_3
UAS_1
UAS_2
UAS_3
Group
UAS
Control
D
Perm Q^2
Perm R^2Y
Frequency
Permutations
Q^2=0.95
p < 0.005 (0/200)
R^2Y=1
p < 0.005 (0/200)
R^2X=0.658

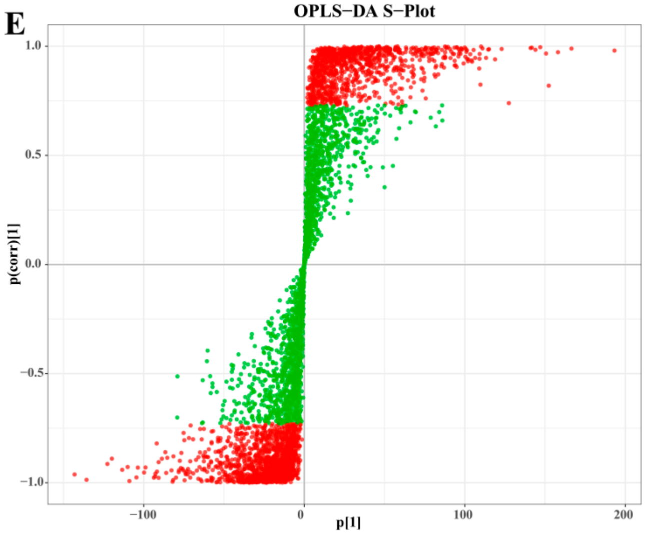

Figure S3. Analysis of the metabolite of chicken soup of different stewing methods: A: PCA plot under positive ion pattern, B: PCA plot under negative ion pattern, C: OPLS-DA plot, D: Permutation test based on OPLS-DA analysis, E: S-plot based on OPLS-DA analysis. Notes: D: Cross-validation results obtained after 1000 permutation tests. E: Change patterns of NVCs with VIP >1 (red) and ≤1 (green) based on OPLS-DA analysis.

A

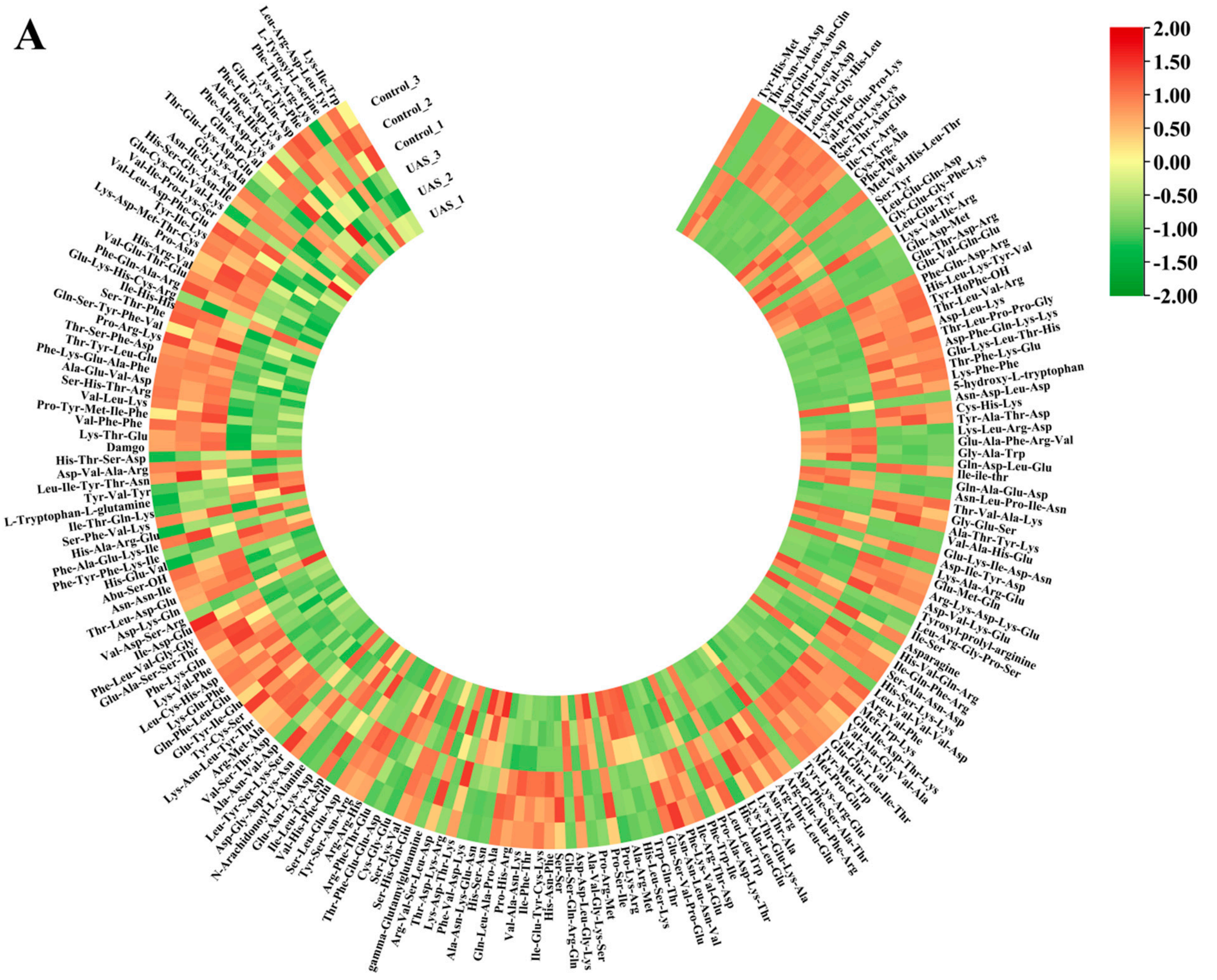
2.00
1.50
1.00
0.50
0.00
-0.50
-1.00
-1.50
-2.00
Control_3
Control_2
Control_1
UAS_3
UAS_2
UAS_1

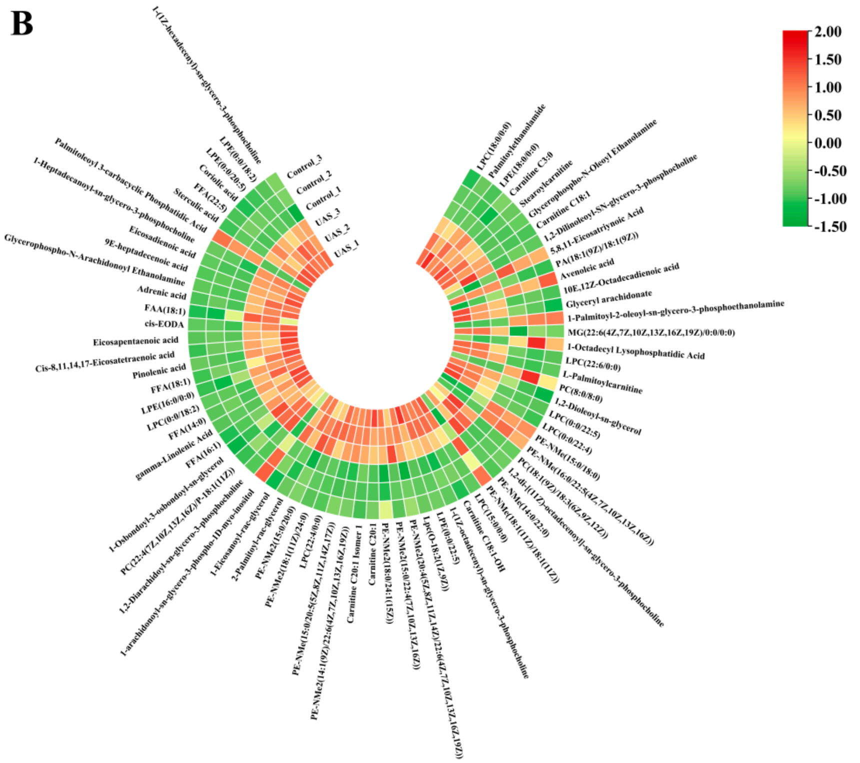
B
Control_3
Control_2
Control_1
UAS_3
UAS_2
UAS_1
1-(1Z-hexadecenyl)-sn-glycero-3-phosphocholine
LPE(0:0/18:2)
LPE(0:0/20:5)
Coriolic acid
FFA(22:5)
Sterculic acid
Palmitoleoyl 3-carbacyclic Phosphatidic Acid
1-Heptadecanoyl-sn-glycero-3-phosphocholine
Eicosadienoic acid
9E-heptadecenoic acid
Glycerophospho-N-Arachidonoyl Ethanolamine
Adrenic acid
FAA(18:1)
cis-EODA
Eicosapentaenoic acid
Cis-8,11,14,17-Eicosatetraenoic acid
Pinolenic acid
FFA(18:1)
LPE(16:0/0:0)
LPC(0:0/18:2)
FFA(14:0)
gamma-Linolenic Acid
FFA(16:1)
1-Osbondoyl-3-osbondoyl-sn-glycerol
PC(22:4(7Z,10Z,13Z,16Z)/P-18:1(11Z))
1,2-Diarachidoyl-sn-glycero-3-phosphocholine
1-arachidonoyl-sn-glycero-3-phospho-1D-myo-inositol
1-Eicosanoyl-rac-glycerol
2-Palmitoyl-rac-glycerol
PE-NMe2(15:0/20:0)
PE-NMe2(18:1(11Z)/24:0)
LPC(22:4/0:0)
PE-NMe(15:0/20:5(5Z,8Z,11Z,14Z,17Z))
PE-NMe2(14:1(9Z)/22:6(4Z,7Z,10Z,13Z,16Z,19Z))
Carnitine C20:1 Isomer 1
Carnitine C20:1
PE-NMe2(18:0/24:1(15Z))
PE-NMe2(15:0/22:4(7Z,10Z,13Z,16Z))
PE-NMe2(20:4(5Z,8Z,11Z,14Z)/22:6(4Z,7Z,10Z,13Z,16Z,19Z))
Lpc(O-18:2(1Z,9Z))
LPE(0:0/22:5)
1-(1Z-octadecenyl)-sn-glycero-3-phosphocholine
Carnitine C18:1-OH
LPC(15:0/0:0)
PE-NMe(18:1(11Z)/18:1(11Z))
PE-NMe(14:0/22:0)
1,2-di-[(11Z)-octadecenoyl]-sn-glycero-3-phosphocholine
PC(18:1(9Z)/18:3(6Z,9Z,12Z))
PE-NMe(16:0/22:5(4Z,7Z,10Z,13Z,16Z))
PE-NMe(15:0/18:0)
LPC(0:0/22:4)
LPC(0:0/22:5)
1,2-Dioleoyl-sn-glycerol
PC(8:0/8:0)
L-Palmitoylcarnitine
LPC(22:6/0:0)
1-Octadecyl Lysophosphatidic Acid
MG(22:6(4Z,7Z,10Z,13Z,16Z,19Z)/0:0/0:0)
1-Palmitoyl-2-oleoyl-sn-glycero-3-phosphoethanolamine
Glyceryl arachidonate
10E,12Z-Octadecadienoic acid
Avenoleic acid
PA(18:1(9Z)/18:1(9Z))
5,8,11-Eicosatriynoic Acid
1,2-Dilinoleoyl-SN-glycero-3-phosphocholine
Carnitine C18:1
Glycerophospho-N-Oleoyl Ethanolamine
Stearoylcarnitine
Carnitine C3:0
LPE(18:0/0:0)
Palmitoylethanolamide
LPC(18:0/0:0)
2.00
1.50
1.00
0.50
0.00
-0.50
-1.00
-1.50

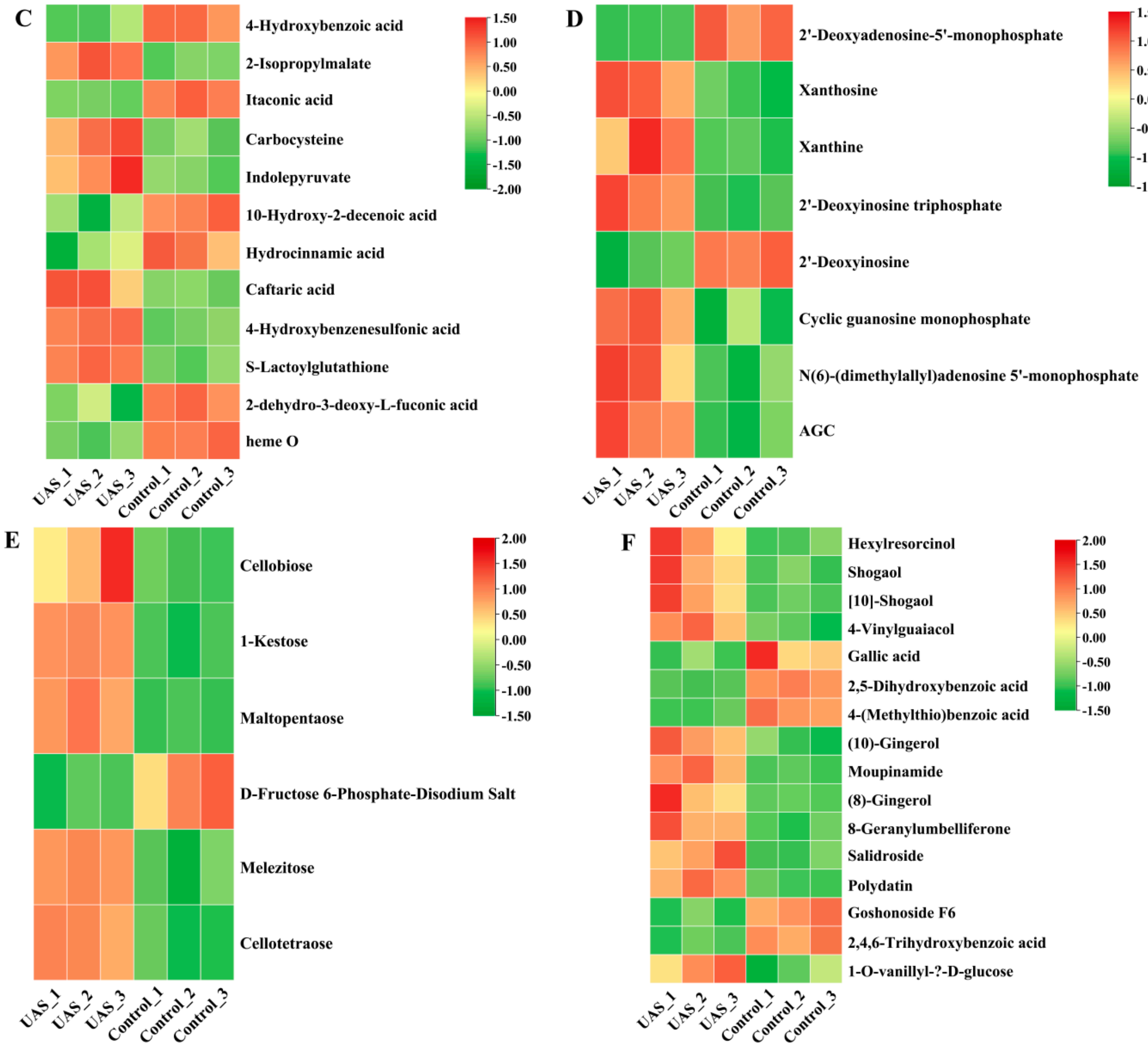
C
4-Hydroxybenzoic acid
2-Isopropylmalate
Itaconic acid
Carbocysteine
Indolepyruvate
10-Hydroxy-2-decenoic acid
Hydrocinnamic acid
Caftaric acid
4-Hydroxybenzenesulfonic acid
S-Lactoylglutathione
2-dehydro-3-deoxy-L-fuconic acid
heme O
1.50
1.00
0.50
0.00
-0.50
-1.00
-1.50
-2.00
UAS_1
UAS_2
UAS_3
Control_1
Control_2
Control_3
D
2'-Deoxyadenosine-5'-monophosphate
Xanthosine
Xanthine
2'-Deoxyinosine triphosphate
2'-Deoxyinosine
Cyclic guanosine monophosphate
N(6)-(dimethylallyl)adenosine 5'-monophosphate
AGC
1.50
1.00
0.50
0.00
-0.50
-1.00
-1.50
UAS_1
UAS_2
UAS_3
Control_1
Control_2
Control_3
E
Cellobiose
1-Kestose
Maltopentaose
D-Fructose 6-Phosphate-Disodium Salt
Melezitose
Cellotetraose
2.00
1.50
1.00
0.50
0.00
-0.50
-1.00
-1.50
UAS_1
UAS_2
UAS_3
Control_1
Control_2
Control_3
F
Hexylresorcinol
Shogaol
[10]-Shogaol
4-Vinylguaiacol
Gallic acid
2,5-Dihydroxybenzoic acid
4-(Methylthio)benzoic acid
(10)-Gingerol
Moupinamide
(8)-Gingerol
8-Geranylumbelliferone
Salidroside
Polydatin
Goshonoside F6
2,4,6-Trihydroxybenzoic acid
1-O-vanillyl-?-D-glucose
2.00
1.50
1.00
0.50
0.00
-0.50
-1.00
-1.50
UAS_1
UAS_2
UAS_3
Control_1
Control_2
Control_3

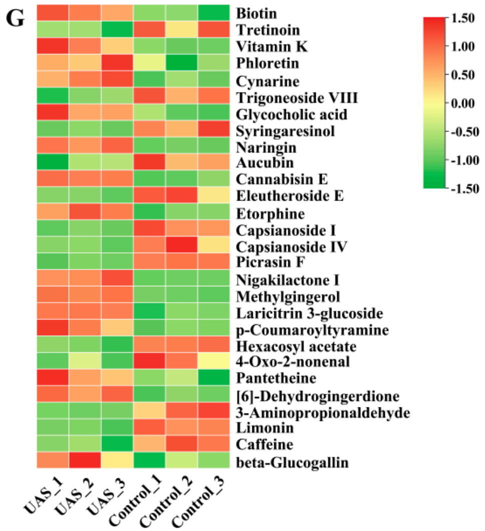

Figure S4. Analysis of the key differential NVCs: A: Amino acids and derivatives, B: Lipids and lipid-like compounds, C: Organic acid and its derivatives, D: Nucleotides and derivatives, E: Carbohydrates and derivatives, F: Benzene and substituted derivatives, G: Others. Notes: Different colors represent different concentrations. (green = low, yellow = moderate, red = high).
